# Understanding the Needs of Moderators in Online Mental Health Forums: Realist Synthesis and Recommendations for Support

**DOI:** 10.2196/58891

**Published:** 2025-09-26

**Authors:** Heather Robinson, Millissa Booth, Lauren Fothergill, Claire Friedrich, Zoe Glossop, Jade Haines, Andrew Harding, Rose Johnston, Steven Jones, Karen Machin, Rachel Meacock, Kristi Nielson, Paul Marshall, Jo-Anne Puddephatt, Tamara Rakić, Paul Rayson, Jo Rycroft-Malone, Nick Shryane, Zoe Swithenbank, Sara Wise, Fiona Lobban

**Affiliations:** 1 Spectrum Centre for Mental Health Research Division of Health Research Lancaster University Lancaster United Kingdom; 2 Division of Health Research Lancaster University Lancaster United Kingdom; 3 Department of Psychiatry University of Oxford Oxford United Kingdom; 4 Berkshire Healthcare National Health Service Foundation Trust Berkshire United Kingdom; 5 Survivor Research Network London United Kingdom; 6 Division of Population Health Health Services Research & Primary Care University of Manchester Manchester United Kingdom; 7 Department of Psychology Edge Hill University Ormskirk United Kingdom; 8 School of Computing and Communications Lancaster University Lancaster United Kingdom; 9 Faculty of Health and Medicine Lancaster University Lancaster United Kingdom; 10 Social Statistics University of Manchester Manchester United Kingdom

**Keywords:** online forums, moderators, psychological well-being, self-determination theory, digital mental health, artificial intelligence

## Abstract

**Background:**

There has been an increase in the use of online mental health forums to support mental health. These forums are often moderated by trained moderators to ensure a safe, therapeutic environment. While the moderator role is rewarding, it can also be challenging. There is a need to understand the impact of the role on moderators and how they can best be supported to maintain psychological well-being.

**Objective:**

This study aimed to understand how, why, and in what contexts moderator well-being is affected by the moderator role and produce actionable recommendations for how moderators can best be supported to maintain workplace psychological well-being.

**Methods:**

We conducted realist synthesis of (1) published and gray literature from 2019 to 2023, (2) stakeholder interviews with forum moderators and hosts, and (3) moderator training manuals developed by organizations that host online mental health forums. Self-determination theory was used as the theoretical basis for this synthesis.

**Results:**

We developed 24 context-mechanism-outcome configurations from our realist analysis of 9 published papers, 18 interviews, and 5 training manuals. The findings highlight the specific ways in which moderator well-being can be supported through meeting the psychological needs for autonomy, competence, and relatedness. Forums that allow moderators to work in alignment with their personal motivations can increase moderator well-being. Forum organizations should support moderator competence through initial expectation setting, especially around moderator responsibility for user well-being, and ongoing support, such as meaningful supervision and peer support. Co-designed training, reflective practice, and experiential learning are all key to increasing moderator competence and satisfaction in the workplace. Working within a diverse team with access to innovative forum design can increase moderator psychological well-being. Organizational support for moderators’ well-being through monitoring and encouraging self-care is vital to ensure moderators can effectively carry out their role. Making and supporting meaningful relationships in the forum can boost psychological well-being and the therapeutic value of the moderator role. Key challenges for moderators were dealing with conflicts between supporting open discussion and ensuring a safe community environment, sharing lived experiences in positive ways for both moderator and user, and supporting people within the limitations of an anonymous forum.

**Conclusions:**

This realist synthesis is the first to examine the impacts on well-being of being a moderator of an online mental health forum. Recommendations to support moderator psychological well-being are proposed, targeted at specific stakeholder groups to aid implementation. Organizational-level endorsement and facilitation of support are particularly important for the realization of recommendations and interventions to support moderator well-being.

## Introduction

### Background

Online support for mental health (MH) problems is growing in line with the increasing demand for digitization [1] and problems with restricted access to health care services [2]. Online MH forums are a type of online support, providing a space for people with MH problems and their loved ones to receive and provide information and support related to MH difficulties [3]. Online MH forums offer 24-7, asynchronous access and the freedom for users to post what and when they choose (within the limits of forums’ terms and conditions), often in an anonymous environment [4,5].

Many online MH forums are overseen by moderators whose primary role is to ensure a safe, therapeutic [6], and engaging [7] environment by (1) providing direct support when needed, (2) managing content through editing or deleting potentially distressing posts, and (3) ensuring safety by identifying and responding to risk of harm to users or other safeguarding concerns [8]. While moderation is acknowledged as an integral role within online MH forums [9] and may have some benefits for moderators, such as satisfaction derived by helping others [8], it is also associated with challenges, such as exposure to distressing content; unclear role expectations; balancing conflicting tasks; and communicating about emotional issues in a limited, text-only environment [10]. Furthermore, the role and associated support needs may be experienced quite differently depending on the moderators’ backgrounds. Some moderators are specifically employed based on their lived experience (eg, the Relative Education and Coping Toolkit trial employed carers of people with severe MH problems as moderators of a forum for carers [11]) and may be motivated to help others in a similar situation [6] by drawing on their shared personal experience to tell relevant stories, normalize experiences, and empathize with forum users. Other moderators are practitioners with formal MH training (such as the registered MH professionals who moderate Togetherall [12]), bringing another type of experience to the role. In other contexts, moderators are employed or work voluntarily without any prerequisite that they have lived experience or formal MH training (such as the volunteer moderators on Reddit [13]). Moderators who draw mainly on their lived experience of MH problems may find that negative emotions are provoked by the personal resonance of potentially distressing content, depending on the support and training they have received. These moderators are often volunteers in a role that may not be supported in the same way as other professional MH support roles, for example, through access to supervision and forums related to guidance and protocols. Given the challenging nature of the role, there is an urgent need to better understand the experiences of moderators in online MH forums and understand how best to support their psychological well-being.

While recent literature has paid more attention to the crucial role of the moderator in online MH forums from the perspective of supporting the forums and their users [3,8] and suggested some ways to support moderators of self-harm and suicide content specifically (eg, broadening practitioner-moderator scope to deal with crisis, provision of timely support, clear guidelines for practice, peer support from other moderators, and multiple channels for interaction with users [4,14]), no research to date has explored the general support needs of moderators of broader online MH forums. Without adequate support themselves, moderators may be less able to offer high-quality support to forum users and more likely to leave their role. As a result, online MH forums may either shut down, leaving users without access to needed information and support, or continue operating with inadequate or no moderation, which can make them potentially unsafe.

### Objectives

The aim of this study was to understand how, why, and in what contexts moderator well-being is affected by the moderator role and how moderators can best be supported to maintain workplace psychological well-being. This was achieved by conducting a realist review of relevant academic literature, synthesized with moderator training manuals and interviews with moderators (conducted by the research team)—hereafter referred to as realist synthesis [15]. The goal was to develop a program theory to explain the key contexts and mechanisms that impact moderator workplace psychological well-being. The findings were used to produce actionable recommendations, based on the program theory, for how support for moderators can be designed, implemented, and iterated in contextually sensitive ways. The study is part of the larger improving Peer Online Forums (iPOF) program to support MH [16].

Realist synthesis is a theory-driven review that draws on a realist philosophy of science to understand contexts and causal mechanisms, which, in this case, explain how and why the moderator role impacts moderator well-being [17]. Self-determination theory (SDT [18,19]) was chosen as the theoretical basis for this synthesis. The definition of well-being according to SDT emphasizes both subjective states, including positive emotion and the significance of functioning well in accordance with personal goals and values [20]. Well-being emerges from the satisfaction of what the theory identifies as universal, basic psychological needs for autonomy (feeling in control of one’s own actions and an alignment between action and personal goals and values), competence (feeling able to carry out one’s role and being effective in doing so), and relatedness (feeling a meaningful connection with others). SDT has well-established, strong empirical support as a framework for understanding well-being, including specifically within a workplace setting (refer to the review by Nunes et al [21]), and therefore provides an appropriate lens through which to explore moderator workplace well-being.

## Methods

Informed by guidance for realist methods [22], the synthesis followed 5 stages.

### Stage 1: Defining the Scope of the Synthesis

At the beginning of the iPOF project [16], the research team—which included mental health academics and clinicians, forum hosts (senior staff with managerial roles at organizations that host online MH forums) and moderators, people with lived experience of MH challenges, and realist methodologists—held a stakeholder consultation workshop. Group discussions highlighted that the demands of conducting moderation are considerable and that a well-supported workforce is essential for the safe and effective delivery of online forums. Improving moderator well-being and retention were identified as key priorities for host organizations. Our initial intention was to focus on the impacts of forums on users only; however, following this consultation, we designed this synthesis to focus specifically on understanding the moderator role and what support was needed to enhance moderator workplace well-being. A parallel realist synthesis conducted by the iPOF team, focused on the impact of forums on user MH, is reported elsewhere [23].

### Stage 2: Developing Initial Program Theories

Initial program theories are preliminary ideas regarding how a process is expected to operate [22]. We built an initial program theory by first drawing on ideas generated through creative brainstorming by the research team based on personal and research experience and through field notes from the initial stakeholder consultation workshop. These initial ideas were mapped onto the psychological needs for autonomy, competence, and relatedness, as defined by SDT. This led to the development of an initial program theory framework tentatively identifying factors that could influence moderators’ psychological needs and therefore their well-being, such as training, supervision, and moderators’ professional backgrounds.

### Stage 3: Searching for Evidence

This synthesis drew on 3 evidence sources: published and gray literature, stakeholder interviews conducted by the research team, and moderator training manuals developed by organizations that host online MH forums.

To identify relevant literature, a systematic search for evidence related to online MH forums was conducted, covering the period from January 2019 to May 2023. The rationale for restricting our search to 2019 was that recent evidence is more likely to reflect the current context in which moderators work within online forums, increasing the relevance of any recommendations made on the basis of this evidence synthesis [24]. This includes website functions, current online culture, trends in use, and the wider context of MH support. Furthermore, initial practice searches suggested that limiting the search to 2019 would mean that the screening burden would be manageable, given the resources available for the review. Databases used to identify evidence sources were Academic Search Ultimate, Embase, Scopus, Web of Science, PsychINFO, MEDLINE, and Allied Health Literature (CINAHL). Search terms were developed with the support of an information specialist at Lancaster University. In addition to database searches, we searched gray literature on Google, Overton, Trip medical database, National Grey Literature Collection, International Clinical Trials Registry, National Health Service Knowledge, ProQuest, and Library Hub. Multimedia Appendix 1 shows the full search strategy.

This synthesis also included an analysis of interviews conducted for the iPOF study [16]. Participants were forum moderators (n=13) and senior forum staff responsible for supervising moderators (n=5), all from UK-based MH forums operated by the National Health Service, charities, and the private sector. Forums were primarily text-based, asynchronous discussion platforms, targeting support for adults and young people (aged >13 years). Participants were recruited through the forum partners as part of the iPOF study via email, provided with written information about the study, and gave written informed consent. These exploratory interviews were consistent with the “theory gleaning” stage of realist interviewing, where key informants provide details regarding how a program operates [25]. Interviewers used a topic guide focused on articulating the impacts of forums, including the role of moderators. As realist evidence synthesis selects only data that are relevant to the development of program theories, only data from these interviews related to the experience of online forum moderation were extracted for this synthesis. Data relevant to the impacts of forums on service users were analyzed for a separate realist synthesis. The topic guide and demographic details of participants are available in Multimedia Appendix 2.

Moderator training manuals were obtained via requests to organizations participating in the iPOF study that host online mental health forums, as well as through Google searches for publicly available documents published by forum providers, which included guidance for moderators on how to perform their role. In total, 5 moderator training manuals were included in this synthesis.

### Stage 4: Selecting and Appraising Evidence

Evidence sources from relevant literature were eligible for inclusion if the evidence referred to forums that met the following criteria:

- The forum included a primarily text-based, asynchronous discussion platform.
- The forum was intended to support user MH, broadly defined to include any issue related to psychological distress. This included forums established to support people with specific MH difficulties, forums aimed at providing general MH support, forums providing support for those caring for someone with an MH difficulty, and forums for those experiencing issues such as loneliness and substance misuse.
- The forum was used by adults and young people, with >50% of users aged ≥13 years.
- The evidence source referenced the experience of forum moderators. Evidence related to forum moderators of any background was included, including paid or voluntary roles.
- No document or study type criteria were applied. Consequently, any document that met these criteria and was available in English was eligible for inclusion.

Titles and abstracts of articles identified by database searches were uploaded to the online systematic review platform Rayyan (Rayyan Systems) [26]. The review team, consisting of 9 researchers, initially screened 100 articles, discussed any discrepancies, and revised the screening procedure as needed. The reviewers were allocated a separate set of articles to screen. When reviewers were uncertain about the eligibility of a source, they flagged it for discussion during the weekly team meeting, where decisions were made collaboratively. The full text of each evidence source that passed the title and abstract screening was then reviewed by a second reviewer to confirm eligibility and appraised with reference to realist informed principles of relevance and rigor [27]. With regard to relevance, evidence sources were eligible if they included data highly relevant to developing program theories about the impacts of moderation. Relevant articles were included if they were also judged to be sufficiently rigorous [28]. Articles were judged as rigorous if their data collection methods were clearly articulated and methods used were consistent with associated results and conclusions. Screening instructions are included in Multimedia Appendix 3 [27,29]. The same initial, large literature search result was used to inform user-focused realist synthesis, reported elsewhere [23]. Each evidence source was therefore screened twice—once to identify content relevant to user impacts, and again to extract evidence related to moderators’ experiences for the purpose of this synthesis.

### Stage 5: Extracting and Synthesizing Evidence

Full texts of documents identified by evidence searches, interview transcripts, and moderator training manuals were analyzed simultaneously. This involved reviewing evidence sources for data related to moderator well-being that could be used to support the development of the initial program theory framework; relevant data segments were copied into an Excel (Microsoft Corp) spreadsheet organized around the 3 psychological needs for autonomy, competence, and relatedness. Codes were added to the data segments to identify important outcomes for moderators, the underlying mechanisms that lead to these outcomes, and what contextual factors were important in triggering these mechanisms. Contextual factors included characteristics of the forums and the individual moderators.

Context-mechanism-outcome configurations (CMOCs) are analytic outputs used in realist research to articulate causal processes that generate specific outcomes related to the process under investigation, which in this case was the moderator role. Following the study by Dalkin et al [30], the mechanism was subdivided into mechanism-resource and mechanism-reasoning. This helps distinguish the part of the underlying mechanism attributable to the program itself (in this case, the moderator role) from the moderators’ responses to that resource (Multimedia Appendix 3 provides details of how CMOCs were operationalized). Initial CMOCs were refined iteratively as the data extraction progressed, with reference to initial program theories, accumulating evidence, and regular discussion within the research team. In the final stages of analysis, CMOCs were explicitly linked to the SDT framework to create an overarching program theory, highlighting how different aspects of the moderator role can support or frustrate moderators’ psychological needs.

Example evidence to support the CMOCs is provided in Multimedia Appendix 4 [3-6,8,14,31-33], but edits have been made to replace any data that identify a specific forum or an individual with a generic term in square brackets. The aim is to protect the anonymity of participants contributing to any of the datasets used.

### Stage 6: Developing Recommendations

We developed recommendations for how moderators can best be supported in the role based on the CMOCs developed from the evidence synthesis and in consultation with key stakeholders, including MH academics and clinicians, forum hosts and moderators, and people with lived experience of MH challenges. Specific and separate recommendations for support were developed for all key stakeholders involved in forum design, implementation, delivery, use, and evaluation.

### Ethical Considerations

This study was granted ethics approval by the Solihull Research Ethics Committee (Integrated Research Application System 314029) on June 20, 2022, as part of the iPOF study [16] and was conducted and reported in line with Realist And Meta-narrative Evidence Syntheses: Evolving Standards guidance [34].

## Results

### Evidence Sources

**Figure 1:**
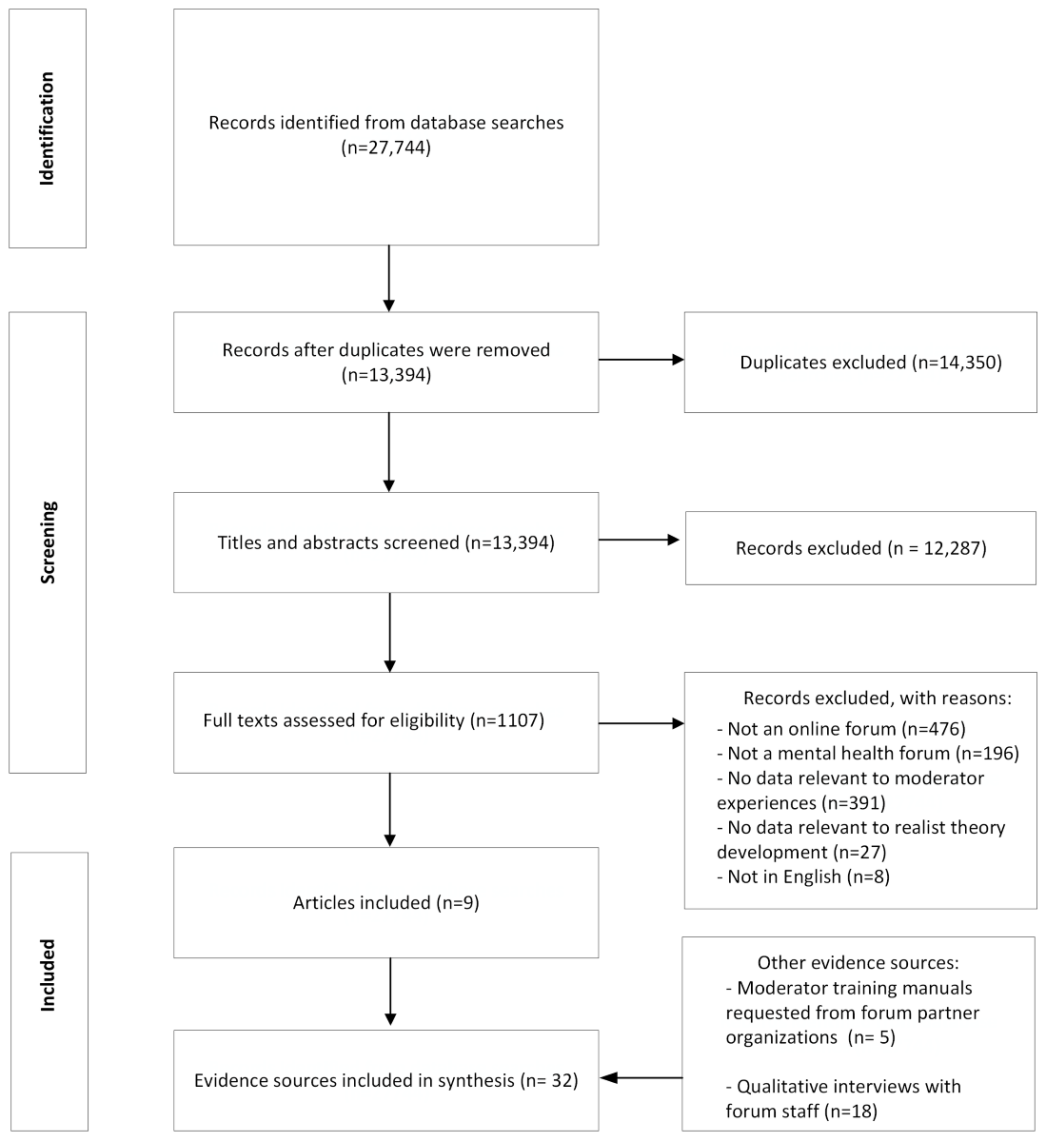
Evidence identification.

We included 9 published academic papers (Figure 1), 13 interviews with forum moderators, and 5 interviews with forum hosts. We also included 5 forum manuals (Multimedia Appendix 5 [3-6,8,14,31-33]) requested from partner organizations. No further gray literature evidence sources were identified.

### Context-Mechanism-Outcome Configurations

#### Overview

We developed 24 CMOCs from our realist analysis, structured around the psychological needs for autonomy, competence, and relatedness. Multimedia Appendix 6 provides a summary of the 24 CMOCs, which together make up the program theory. The narrative summary in the subsequent sections, along with Multimedia Appendix 6, should be read with reference to Multimedia Appendix 4, which provides quotes from the data in support of each CMOC. Finally, based on the CMOCs, Table 1 presents recommendations of ways in which moderators can be supported to achieve workplace psychological well-being.

**Table 1:** Key recommendations for supporting moderators of online mental health forums.

Stakeholder group	CMOCs^a^	Recommendations
For forum hosts	3-15 and 17-19	Forum hosts have a broad responsibility for enhancing moderator well-being and for facilitating them to effectively carry out the role. Hosts should do the following: Consider the value of payment for moderators. Prewarn moderators of exposure to potentially distressing contact and ensure they are aware of the support available to them. Monitor moderator well-being and provide access to support, such as individual reflective supervision and peer support in a safe space that fosters trust and openness. Provide training that is personalized to the culture and content of the specific forum and co-designed with (and delivered by) people with experience of moderation. Allow space within training for shadowing more experienced moderators, exposure to the forum, and practice shifts and feedback. Remind moderators of what is realistic in terms of how much control they have over users’ situations. Support moderators to respond to user posts with a unified voice in line with the forum culture. Provide evolving, up-to-date guidance on how to identify and respond to risk appropriately. Provide training focused on developing mental health literacy related to the specific mental health experiences likely to be discussed on the forum. Provide access to resources that enhance moderator knowledge and expertise, such as trusted information and services. Ensure moderators work as part of a team with diverse skills and experiences. Provide support to help moderators manage competing demands on their time. Encourage moderators to monitor their own well-being and implementation of self-care strategies (a valuable ethos may be “you can’t support others if you don’t support yourself”).
For forum designers	14-16	Forums should be designed in such a way that moderators are supported to do their job. Forums could be designed to include the following: A function for moderators to message a user privately and directly. A function to flag potentially problematic posts for continued monitoring. Access to interaction mechanisms other than post-comment based, such as emojis.
For forum moderators	4-12 and 17-24	Forum moderators should do the following: Familiarize themselves with the role and what to expect before commencing the role of a moderator. Engage in ongoing training provided. Reflect on and refine their practice in line with feedback from supervision, other moderators, and forum users. Take care to ensure expectations are realistic regarding how much control they have over a user’s situation. Signpost users to host-approved up-to-date resources when appropriate. Draw on the diverse skills within the team. Monitor their own well-being. Implement personalized self-care strategies as needed. Reach out for support from other moderators, supervisors, and hosts as needed. Proactively moderate to collaboratively shape the tone and community environment. Be mindful of when their input is needed and when it could impede peer support, that is, by “jumping in too soon” and preventing users from connecting with one another. Ensure everyone gets a timely response, particularly if they are new users or their post indicates risk. Take care to ensure professional boundaries are maintained. Ask open questions to find out more about a user’s situation if it is not clear. Draw on lived experience, where applicable.
For forum team members, for example, other moderators	5, 12 , and 17	Forum team members should do the following: Try to recognize and monitor the challenges moderators face. Be available to provide support and guidance (eg, reiterating role boundaries and expectations for support provision), particularly when other moderators are struggling.
For forum users	1 and 17	Forum users should do the following: Try to provide positive affirmation whenever appropriate—a thank you from a user goes a long way! Recognize the emotional challenges associated with moderating (eg, they cannot please everyone all the time). Try to be sensitive to the needs of the moderators as well as the forum community.
For researchers	1-24	Future research should aim to do the following: Refine the theories. Explore the impact of the recommendations on moderator (and user) well-being in online mental health forums. Test the transferability of recommendations to general health forums.

^a^CMOC: context-mechanism-outcome configuration.

#### Autonomy (Feeling in Control of One’s Own Actions and an Alignment Between Action and Personal Goals and Values)

Where moderators’ motivations for engaging in the forum align with the role requirements, the psychological need for autonomy is more likely to be met (CMOCs 1-3). A key motivation for moderators to take up the role was to support others. Moderators felt a sense of accomplishment when they witnessed supportive communication and success stories on the forum because they interpreted this as evidence that their work had been beneficial in supporting users (CMOC 1). Moderators may experience distress when their required duties conflict with their personal motivations, for example, wanting to support the needs of users. A particular example of conflict was between respecting the needs of an individual user sharing a sensitive post versus protecting the wider forum community from harm through exposure to distressing content by editing or removing a post (CMOC 2). Where payment is the norm in specific organizational contexts, moderator motivation may be increased through payment congruent with the demands of the role. Payment may make moderators feel valued and decrease perceptions of the need for additional paid work, increasing moderator motivation and the likelihood of retention (CMOC 3).

#### Competence (Feeling Able to Carry Out One’s Role and Being Effective in Doing So)

On commencing the role as a moderator, some feel a lack of competence and worry they might “say the wrong things and make someone’s day already worse than they were feeling” (moderator 9). Increasing moderator competence to fulfill the requirements of the role is likely to increase workplace psychological well-being and can be done in several ways, as outlined in the subsequent sections (CMOCs 4-18).

##### Increasing Competence Through Initial Expectation Setting and Ongoing Support

Moderator competence can be increased by improving their understanding of the role from the outset and providing support to help them manage the ongoing emotional impact of exposure to potentially distressing online content (CMOCs 4 and 5). Before commencing the role, a clear outline of what moderators can expect from the role, in terms of exposure to distressing content and access to support, allows moderators to decide if or to what extent they are able to moderate (CMOC 4). Access to ongoing, regular reflective individual supervision and peer support, such as teaming up with (buddying), observing (shadowing), or informally chatting with or meeting (informal daily support) another moderator or moderators provides an opportunity for joint decision-making and reflection on how to manage challenging posts. Supervision and peer support also allow a space for refinement and validation of practice in line with feedback. All of this diffuses feelings of individual responsibility for user well-being and increases feelings of support (CMOC 5).

##### Increasing Competence Through Training

Moderator competence can be increased through forum-specific, comprehensive, ongoing training (CMOCs 6-10) that includes shadowing more experienced moderators, exposure to the forum, practice shifts, and feedback so that moderators feel clear about how to moderate and less worried about making a mistake (CMOC 6). Training is more likely to be effective at building competence when it is co-designed with (and delivered by) people with experience of moderation and detailed understanding of the specific forum culture and the challenges the moderators are likely to face because moderators understand the relevance and applicability of the training information provided (CMOC 7). Ensuring that moderators do not feel isolated or overwhelmed by their responsibility for user safety and well-being is an important aspect of training (CMOCs 8 and 9). This can be achieved by (1) managing moderator expectations about how much control they have over a user’s situation, given the constraints of some anonymous online forums (CMOC 8); for example, emphasizing that the lack of control moderators have over what users do, that their role is to provide guidance and support, and that their supportive responses may be comparatively much faster than waiting for access to face-to-face services, and (2) providing evolving, up-to-date risk protocols so that moderators feel competent and confident to identify and respond to risk without making a mistake in an already high-risk situation (CMOC 9). Risk protocols may be especially important for moderators in an online environment where the potential for risk-related posts is high and nonverbal cues are removed. They are also critical for MH service staff who are used to working with established protocols and may be concerned about the impact on their professional status if protocols are not provided (CMOC 9). Where applicable, training should include a specific focus on increasing MH literacy for moderators with limited MH experience in relation to the specific forum content, so that moderators feel more prepared and comfortable to respond to users’ personal situations (CMOC 10).

##### Increasing Competence Through Access to Resources

Moderator competence is increased through access to resources that enhance knowledge and expertise (CMOCs 11 and 12) and decrease burden (CMOC 13). Specifically, access to host-approved, up-to-date resources (eg, psychoeducational information from reputable sources) and the contact details of relevant services allows moderators to more easily respond to posts outside their area of expertise because they feel reassured by the option to signpost users to trusted alternative resources (CMOC 11). Working within a team of moderators with diverse skills and experience decreases moderator anxiety around responding because they do not feel individually responsible for user well-being and more supported to provide a collaborative response (CMOC 12). However, moderators may find it tough to fulfill conflicting responsibilities where they have another role, such as an MH professional delivering in-person services, and the associated responsibilities conflict with forum moderation. In this context, there is a risk that moderation will be deprioritized (CMOC 13).

##### Increasing Competence Through Forum Design

Forums should be designed in a way that supports moderators to do their job effectively (CMOCs 14-16). Having a private space to communicate with individual users directly may enhance moderators’ ability to communicate openly and in a more personalized style because they feel comfortable doing so, which may be especially helpful where the content is sensitive or personal (CMOC 14). However, it has been suggested that moderators should be cautious not to encourage users away from the peer support offered on the forum and toward dependency on the moderator for support (CMOC 14). Well-designed systems that include design features outside the post-comment text-based format, such as emoji responses, allow moderators to focus on more detailed text-based responses because they feel supported by the community in providing appropriately matched support to other users (CMOC 16). The option to flag potentially problematic content for continued monitoring can also enhance moderator ability to meet the demands of the role because moderators feel able to respond quickly if there is a need to intervene (CMOC 15).

##### Increasing Competence by Supporting Moderator Well-Being

It is vital that forum hosts are aware of moderator well-being and make efforts to ensure well-being is supported so that moderators have the capacity to fulfill the role (CMOCs 17 and 18). Moderator resilience to the emotional impact of the role fluctuates depending on their own levels of well-being. Where moderators are in the right frame of mind, familiarity with forum content through repeated exposure or lived experience can increase moderator resilience to the emotional impact of moderating distressing posts. However, repeated exposure to distressing content can lead to desensitization if moderators do not make efforts to ensure that this does not happen (CMOC 17). Forum hosts should provide moderators with appropriate measures to ensure their own well-being so that they feel supported to engage in personalized self-care strategies to minimize the negative impact of forum moderation. Self-care strategies, such as taking regular breaks, not working overtime, reaching out for support, and boundary setting (time, location, and role), are particularly important for moderators working from home. These strategies are important in all forums but are particularly necessary in forums related to suicide and self-harm or where forum content can be distressing and may have high personal resonance (CMOC 18).

#### Relatedness (Feeling a Meaningful Connection With Others)

Moderators are more likely to experience workplace psychological well-being when they make or support meaningful connections within the forum (CMOCs 19-24).

##### Supporting Users to Connect With the Forum and Each Other

One of the main responsibilities of moderators is to facilitate user engagement with the forum and with each other by creating “a space where conversations can happen” [31]. By giving moderators guidance on response styles and how to enforce rules, for example, by editing text; requesting users to use language more appropriate to the forum culture; or modeling helpful posts, moderators better understand how to collaboratively shape the tone and community environment in line with the forum’s culture, producing a supportive, productive, and judgment-free community (CMOC 19). Our analysis highlights the importance of moderators being supported to understand when it is safe to avoid responding to a user’s post so that they are able to judge when their input is needed (eg, when a post is from a new user or it has not received a response) and when it could impede support, that is, by “jumping in too soon” and preventing users from connecting with one another. This creates an opportunity for the forum to become self-reliant, connected, and engaged (CMOC 20), which is particularly important for the provision of support when moderators are not online.

##### Making a Connection With Users

Developing connections with users can be very rewarding for moderators and can facilitate their ability to carry out the role; however, such connections can also make the role more challenging in terms of the impact on moderator well-being. Connections can be facilitated by moderators drawing on their own lived experience (if appropriate) and that of the community, but this requires careful navigation in terms of how and when this is done (CMOCs 21-24). Moderators often get to know users over time through the stories they tell and develop meaningful connections; therefore, it is important that moderators are supported to identify personal and professional boundaries to avoid potentially unhealthy dependency of users on moderators and worry about users on a personal level (CMOC 21). By exploring users’ experiences and needs for support through asking open questions to invite users to expand their post, moderators can reduce any anxiety associated with not knowing what is happening for the user and increase the likelihood that their response matches user needs and is therefore helpful because they feel more knowledgeable about the users’ situation (CMOC 22). Complete anonymity may promote the disclosure of distressing experiences but also limits the support and follow-up moderators are able to offer, which can lead to moderators feeling disempowered and uncertain about user safety, which undermines moderator well-being (CMOC 23). One way to provide effective support is by drawing on shared lived experience; by drawing similarities with a user’s situation, moderators can provide an authentic, empathetic response based on personal experience and a detailed understanding of the user’s mental illness experience (CMOC 24).

## Discussion

### Principal Findings

This realist synthesis is the first to explore the impacts of being a moderator of MH online forums on moderator well-being. The review highlights ways in which moderator autonomy (CMOCs 1-3), competence (CMOCs 4-18), and relatedness (CMOCs 19-24) are linked to workplace psychological well-being. The findings have clear and direct implications for practice, which are explored in the Implications for Practice section and summarized in Table 1. Recommendations are organized by stakeholder group to support targeted and effective implementation.

### Alignment With Previous Research

Feeling in control of one’s actions and that those actions align with personal goals, values, and motivations is important for supporting the psychological need for autonomy. We found that working in a way that specifically aligned with personal motivations was important for moderator psychological well-being. Contexts in which people are free to be autonomous promote motivation [35], which in turn increases the likelihood of retention [36]. We found that a key motivation for moderators was to support others and that where evidence, such as supportive communication and success stories on the forum, was interpreted as this motivation being met, moderators felt a sense of satisfaction. In addition, feedback from supervisors and peers through reflective supervision, peer support, and training is important for enhancing moderators’ ability to carry out the role effectively, thereby increasing the likelihood that their motivation to support others is met. Feedback from an organizational level, as well as adequate payment, may also demonstrate value for the moderator role and the challenges associated with it. Indeed, organizational-level feedback has been shown to be important for well-being in other health-related workplace settings [37].

Positive feedback (whether explicit or interpreted through positive forum content) also leads people to feel effective [38], which is a key factor underlying the psychological need for competence [19]. Competence has been found to be foundational to workplace well-being in the broader organizational literature [39]. Our findings align with previous research in highlighting the importance of recognizing the emotional needs of employees and providing support through ongoing, regular, individual supervision [40], which provides a space for moderators to explore what is expected of them, discuss challenges, and refine practice. A particular parallel with previous findings was the importance of aligning support and training with the organizational culture and forum content and reflecting key stakeholder views through co-design to increase the relevance and applicability of information provided. The incorporation of experiential learning and peer support into training also emerged as important to enhance moderator ability to understand and respond to the more dynamic demands of the role and feel less worried about making a mistake (which would undermine the goal of supporting others). In similar roles, such as peer support workers, the importance of peer support has also been highlighted [41]. For moderators working online, peer support, such as buddying and informal contact with other moderators, may be particularly important to reduce feelings of isolation, especially when moderators are working from home.

A key threat to moderator competence was holding unrealistic perceptions of how much control they have over a user’s situation within the context of an anonymous online forum. Indeed, role ambiguity and conflicting role boundaries have previously been linked to poor employee MH [42]. We suggest that this threat to moderator competence can be reduced by setting clear expectations early regarding role responsibilities and the limits of personal responsibility for user well-being. In addition, ensuring moderators work within a diverse team can help them assess their capacity to moderate, manage unrealistic expectations, and feel less solely responsible—while more supported—in providing a collaborative response. A further threat to moderator efficacy also highlighted in the literature [6] is the increasing burden placed upon them as communities grow. Adequate staffing may go some way to alleviating this threat, but we suggest this could be bolstered by innovative forum design, such as alternative response formats (eg, emojis), which are quicker and, in some cases, more appropriate to implement. This allows moderators to focus their time and attention on posts that require more detailed, text-based responses. Implementing these features when user posts do not require a tailored response could aid scalability of forums by decreasing moderator burden.

In line with broader organizational literature [43], we found that forming positive relationships with other moderators in a forum context satisfies the psychological need for relatedness. Forging meaningful relationships with coworkers in the same role has also been shown to prevent mental ill-health in a health care context [37]. Moderators’ relationship with users may provide another avenue for enhancing the psychological need for relatedness, particularly for those who have been moderating the same user group for a prolonged period. However, we found that it is important to support moderators with identifying personal and professional boundaries so that they avoid potentially unhealthy dependency while maintaining positive relationships with users. We found that drawing on lived experience, where their specific role facilitates the disclosure of advice and support based on those experiences, could lead to personal satisfaction, but that there may be complexity around decisions related to how and when to share, which is recognized elsewhere in the peer support literature (eg, Gillard et al [44]). We suggest that the psychological need for relatedness could also be met by going beyond developing relationships with others, as posited by SDT [45], to include facilitating relationships between others, in this case between forum users. Moderators may derive satisfaction and enhanced well-being from cultivating and witnessing a positive, supportive environment in which users support each other. Therefore, it is important that moderators are given guidance to ensure they understand how to collaboratively shape the community environment to be a positive space and when it is safe to avoid responding to allow the forum to become self-reliant.

### Implications for Practice

Moderated online MH forums are potentially a very valuable way to support people and have great potential. However, uncertainties regarding the moderator role and how to best support moderators may make organizations reluctant to host a forum, moderators unsure about taking on the role, and users unclear about the support they provide and therefore the safety of the forum [23]. On the basis of our theories, we have proposed some key recommendations for supporting moderators of online MH forums for different audiences (Table 1). Implementation of these recommendations requires significant buy-in at an organizational level [37,40] and commitment to cultivating a supportive environment in which the ethos is for everyone involved in a forum to be aware of, and supportive of, moderator well-being. Supervisors need to be familiar with forums, which requires training from the organization. Moderators should be trained in relation to the specific forum they are working on, which requires endorsement by the organization. As detailed elsewhere [16], we have developed co-designed best practice guidelines for moderator training, but support from the organization is key for these guidelines to be effectively implemented. All recommendations require modification to align with the specific forum contexts, such as forums involving lived experience moderators or forums in which moderators work from home.

### Limitations and Future Directions

There are some limitations to this research. Interviews were conducted with stakeholders recruited from UK-based MH organizations; therefore, their experiences may not represent those of individuals in other locations, settings, and cultural contexts. Although we have paid particular attention to the importance of context, we acknowledge that context is constantly changing, particularly around perceptions of who holds responsibility for what happens in forums. For example, in the United Kingdom, the Online Safety Act [46] requires platforms to ensure that children are prevented from accessing harmful content, such as content encouraging suicide, self-harm, or eating disorders. The emphasis on ensuring safety and removing potentially distressing content in forums is likely to increase —along with the associated pressure on moderators. Therefore, this work is timely in exploring ways we can support moderators to thrive and continue to keep forums safe.

Our findings call for further realist research to evaluate and refine the program theories proposed. This should be done with reference to impacts on moderator well-being and the indirect impacts on user well-being to ensure that there are no unanticipated negative impacts for users. To our knowledge, there are no workplace well-being interventions for moderators of online MH forums, despite the importance of this role and increasing demand for online support; therefore, this is an important focus for future work. Future research should also consider the potential of artificial intelligence tools to manage some of these tasks, which will change the role of moderators.

### Conclusions

Online MH forums are increasingly being used to support MH and are often moderated by trained moderators to ensure a safe, therapeutic environment. The moderator role is rewarding but can also be challenging. Moderator well-being can be supported by meeting the psychological needs for autonomy, competence, and relatedness, for example, by allowing moderators to meet personal motivations; supporting moderators’ emotional and practical needs through supervision, peer support, and co-designed training; and supporting the formation of positive, meaningful relationships with other moderators, with users, and among users. Organizational-level endorsement of support and the cultivation of a supportive organizational philosophy are particularly important for the realization of recommendations and interventions to support moderator well-being. However, the role is likely to change in response to increased forum use, increased demand from society for hosts of online spaces to take responsibility to keep people safe, and the developing role of artificial intelligence in forum moderation. Further research is needed to test the effectiveness of the recommendations proposed while recognizing the need for constant refinement as the landscape of online MH forums continues to change.

## Appendix

Multimedia Appendix 1 Search strategy.

Multimedia Appendix 2 Interview participant demographics and topic guide.

Multimedia Appendix 3 Full-text screening instructions.

Multimedia Appendix 4 Example evidence.

Multimedia Appendix 5 Included evidence sources.

Multimedia Appendix 6 Context-mechanism-outcome configurations.

## Abbreviations

CMOC: context-mechanism-outcome configuration
iPOF: improving Peer Online Forums
SDT: self-determination theory
