# Supplementary material for: Understanding the Needs of Moderators in Online Mental Health Forums: Realist Synthesis and Recommendations for Support
Source: JMIR Ment Health. 2025 Sep 26;12:e58891. doi: 10.2196/58891 (PMC12514405; doi:10.2196/58891)
Supplement: Multimedia Appendix 4 [file mental_v12i1e58891_app4.docx]

# Examples of the data that provide evidence for the CMOCs

Examples of data that provides evidence for the CMOCs are provided here, but edits have been made to replace any data that identifies a specific forum or individual with a generic term in square brackets. The aim is to protect the anonymity of participants contributing to any of the data sets used. Moderator and host demographics can be seen in Appendix 2.

Autonomy (feeling in control of one’s own actions and an alignment between action and personal goals, values and motivations)

## CMOC 1: Alignment of moderation with personal motivation to help others

If moderators are motivated to support others, perhaps due to previous similar personal experience, (C) then they will feel a sense of accomplishment and satisfaction (O) from seeing supportive communication and success stories on the forum (M – resource) because they interpret this as evidence that their work has been beneficial in supporting forum users’ well-being (M – reasoning).

### Example evidence

C

Several moderators described their intention to moderate being **driven by a desire to help others**: “I’m sort of in this line of work ‘cause I want to help people”. For some, this desire came from a general interest in mental health, whereas others had a particular interest in self-harm, **often due to their personal experiences**. [1]

“I like helping people but also I am very much **driven by wanting other people to have better experiences than I did** growing up and making sure that they get support in a timely way that feels safe for them which I didn’t feel that got growing up, so **very much motivated by personal lived experience**.” Moderator 12

...most of our participants **agreed to moderate solely on altruistic reasons**. They answered that it does feel good in helping fellow members undergoing mental health challenges. One participant attributed moderation as a form of “community service”, and an excerpt of interview response demonstrating altruistic reasons, goes like, **“I have gone through depression myself. Knowing what depression can do to someone, I aspire to offer support where I can because nobody deserves to feel so low.** Being able to support others also greatly improves my self-confidence and overall makes me feel better about myself. This motivates me to support more people.” [2]

M

**“We’re seeing amazing success stories**. I’ve had users that I’ve worked with over long periods of time and I’ve seen them go from being in absolute dire straits to one of them is now a mum herself and she is training to be a social worker and so when you actually work with young people for a long time and you hep to facilitate that peer support and we also have live forums which are kind of like online workshops where we do quite a lot of [psycho] education, so **when you then see them putting those skills that you’re teaching them into practice and then telling other young people, ‘Hey this coping mechanism really works for. Have you tried it?’ you really see how beneficial your work is**” Moderator 12

O

**Moderators get a lot of satisfaction from watching friendship form between users**, especially given the restrictions of the online environment on making intimate connections [3].

“**expressed the sense of accomplishment when seeing members making satisfactory progress towards recovery** and mentioned feeling inspired by the tremendous amount of resilience some members have demonstrated” [3]

## Evidence sources

[1]

[2]

[3]

Moderator 8

Moderator 10

Moderator 11

Moderator 12

Moderator 13

Moderator 16

Forum manual 1

## CMOC 2: Conflict in personal motivations

When users post content that is potentially distressing for the community (C1), and moderators are required to edit or remove content (M-resource1) this can cause moderators’ discomfort (O1), because they are conflicted between wanting to respect the needs of the user wanting to share and ensuring the forum does not become a sanitised space versus wanting to protect the community from exposure to potentially harmful material (M – reasoning1).

### Example evidence

C

The [moderators] sometimes mentioned the discomfort they can experience if altering members’ posts, while acknowledging that it is **necessary to do so in instances where these messages might potentially be distressing for other members.** [3]

M

The [moderators] described the way in which managing these scenarios, while not frequent among members, still **requires meticulous attention in order not to distress the rest of the community.** At the same time, **having to alter certain member’s posts to prevent harm to the community is reported to be among the biggest challenges for a [moderator]. Most find it hard to gauge the right level of modification: seeking to balance staying true to the original post and permitting freedom of expression “allowing the community to breathe”; yet, still avoiding community harm.** [3]

“The biggest problem was people posting very traumatic stuff that sometimes erects what is thought might be very upsetting for other people to read and that was quite tricky because **we didn’t want to make it a sanitised space** that nothing real was happening in but equally graphic detail of suicide attempt or something could be very upsetting.” Host 5

O

The [moderators] sometimes mentioned the **discomfort they can experience if altering members’ posts,** while acknowledging that it is necessary to do so in instances where these messages might potentially be distressing for other members. [3]

“I think this is one of the biggest challenges for me because **it feels so invasive**. They very specifically have chosen words that express something inside whether it is intentional or whether it is a process of intent. Their intent is very seldom to hurt anyone else and so when we edit, we do that because we let them know that it could actually hurt someone else. And even though we know that that's not the intent, and even though they know we know that it, I think it is incredibly shaming, and **I find that part of the process really, really, difficult.**” [participant] [4]

## Evidence sources:

[1]

[3]

[4]

[5]

Moderator 6

Moderator 10

Host 3

Host 5

CMOC 3: Alignment of moderation with expectation for payment

Where payment is expected within the specific organisational context and of adequate value to the individual moderator (C), payment for moderation (M – resource) may help moderators feel happy and satisfied that their contribution to the forum has been recognised and is valued (M – reasoning1) and decreases moderators’ perception that they will require additional paid work (M – reasoning_2_), increasing their motivation and the likelihood of retention (O).

### Example evidence

C

because of the things they see, the things they have to moderate. ...I think you do a job where you’re helping people and you have hours and things like that yeah **you should be paid for doing that**. Whatever your intentions, even if it’s just you’re doing it for a job or whether like me you have a personal investment **it’s a job and jobs should be paid.** Moderator 12

M

“It’s because also if you’re doing moderation in any form you still need to pay the rent, you still need to pay for food and things like that so **most people that are moderating out there in the world are doing it on top of jobs**. This is stuff they’re doing in their own time so the hours they end up working every week to make sure they can pay their bills and also moderate and it is almost more like a hobby thing for them but with added occasional emotional trauma.” Moderator 12

“and in an ideal world **if it was really valued we’d be saying ‘Look we really value your time and your moderation and we’d be willing to pay you for it as well,**’ so kind of a part of what we’re offering as a service but we’re not at that stage yet.” Moderator 3

“but when I first started you were just in the background, nobody really knew who you were just the moderators and it wasn’t a very valued role, a **very underpaid role as well for the stuff that we were dealing with**” Moderator 2

“I mean apart from the fact that **it helped me pay my bills** it was just incredible because **I actually felt taken seriously and like a professional in my own right** you know.” Moderator 12

O

It does mean a lot to me specifically. I think there are people that would go, ‘Well yeah you’re doing a job like of course, why wouldn’t you expect to get paid?’ but having done it for a very long time without **it is something I think is pretty amazing**.” Moderator 12

## Evidence sources:

Moderator 2

Moderator 3

Moderator 12

## CMOC 4: Moderators are more able to carry out the role effectively when they know what to expect

Moderation can involve exposure to descriptions of potentially highly distressing experiences, such as self-harm (C). Being prewarned of exposure and informed of support available within the team in initial training (M – resource) can help moderators decide (M– reasoning) if or to what extent they are able to moderate at that time (O).

### Example evidence

C

“I was prewarned of the job role because it’s health, you can get **quite a few people who mention suicidal tendencies or self-harm** because some of them for example are in so much pain, they’ve given up or they feel let down by their GP about their mental history”. Moderator 11

M

“**I was prewarned of the job role** because it’s health, you can get quite a few people who mention suicidal tendencies or self-harm because some of them for example are in so much pain, they’ve given up or they feel let down by their GP about their mental history”. Moderator 11

“I mean obviously **we train them at the beginning and quite a lot** – so there were a range of elements to that. I mean it was very clear that there job wasn’t to provide advice.” Host 2

O

If a potential [Forum moderator] is overwhelmed by their own current caring responsibilities then this role might not be for them at this time. However, the nature of the role, means that they will sit within a team able to offer understanding, support and guidance when personal caring situations fluctuate. **Deciding whether this is the right time** for an individual to become a RS **will depend on assessing how it fits with individual personal situations and the support systems offered by the team**. Forum manual 1

## Evidence sources:

Moderator 11

Host 2

Forum manual 1

CMOC 5: Moderators feel more able to carry out the role effectively when they are supported by supervisors and peers

If moderators need support with difficult issues related to forum content or specific users (C) and engage in reflective supervision and peer support in a safe space that fosters trust and openness (M-resource) then they will be more competent to deal with the issue (O) because they feel supported, to learn and less isolated in their responsibility for user well-being (M-reasoning).

### Example evidence

C

Feeling really heard by a supervisor is as important as an [Forum moderator] feeling comfortable about **raising difficult and important issues** **that might be of an intensely personal nature to them**. Forum manual 1

“Basically, I can book in with one of our trained supervisors at least once a month, sometimes more if I need it and get an hour or like I say if I need more and I can just talk to someone in confidence about **if I’m struggling with a specific service user or how to work with them or certain themes that we’re seeing on the site**…. Moderator 12

M

“I think you need someone who’s looking after the moderators and who is available and that they have the paid **time and space to really reflect and share** and that kind of thing” Host 5

**Feeling really heard by a supervisor** is as important as an [Forum moderator] **feeling comfortable** about **r**aising difficult and important issues that might be of an intensely personal nature to them. A supervisor will not know about an issue that has not been raised with them, therefore **developing trust and a good working relationship is an important part of a successful supervision**. Forum manual 1

“Basically, I can book in with one of our trained supervisors at least once a month, sometimes more if I need it and get an hour or like I say if I need more and I can just talk to someone in confidence about if I’m struggling with a specific service user or how to work with them or certain themes that we’re seeing on the site….or things like if something’s come up in our personal lives so when I lost my friend to suicide I sought supervision because I was like, ‘I know that this is unavoidable seeing posts about this at [forum] so what would you recommend? Can we just talk it out? Can I spend a bit of time just talking about him and what he meant to me and that loss?’ so **it is a therapeutic space for us**.” Moderator 12

“I think we sort of call it **reflective practice** so it’s just a space for them either one on one or as a group to just reflect on difficult situations and just have a bit of support from a trained therapist.” Moderator 1

“Something we do want to do is **create a buddy system so they have two people who are on together so it’s not so isolating** and that was something that came out – we did a bit of research with another uni before and that was something that came out of interviews with our moderators.” Moderator 6

“**Having support from an experienced peer moderator may provide increased opportunities for learning.**” Moderator 5

The other thing I think is really important is the [forum name] supporters here supervision between them was really important so although [name and name] facilitated it I think they actually got more – not more, something, **a lot from talking to each other and checking out things with each other.** Host 5

O

“We had a lot of buddying sessions so they are training sessions that we had and it was very helpful because I had someone who is very experienced in moderating and in giving advice to other people like training me and I thought, ‘Wow, this is actually not too bad.’ You get out of that meeting **feeling much more like it’s easier to do something because you understand exactly how to word your phrases or how to offer pieces of advice** in this area and a lot of them are so incredibly helpful.” Moderator 4

## Evidence sources

[1]

[6]

Moderator 1

Moderator 3

Moderator 4

Moderator 5

Moderator 7

Moderator 12

Moderator 13

Host 2

Host 5

Forum manual 1

Forum manual 2

CMOC 6: Moderators feel more able to carry out the role effectively when they have space and time to practice

Where moderators are given time to build and practice new skills (C), training that involves shadowing more experienced moderators, exposure to the forum, practice shifts and feedback (M-resource) increases moderator’s feelings of competence to moderator alone (O) because they feel clearer about what how to moderate and less worried they will make a mistake (M-reasoning).

### Example evidence

C

Although some participants mentioned training (through The Mix or, often, elsewhere) as a way of developing their skills, **practice and experience** were considered by all as the most effective way in becoming competent. [participant] felt “You’ve got to have quite a bit of experience….if you haven’t seen other posts, you can’t really know”. [participant] added, “**there was a lot of practice shifts first which was great**”. This practice allowed moderators to receive feedback. [1]

Nobody comes with a full toolkit. I don’t think anybody ever has the full toolkit. We’re always learning but I think at the beginning it is a much steeper learning curve but I think **it’s about building your confidence and taking your time. There is no pressure.** Moderator 13

M

Although some participants mentioned training (through The Mix or, often, elsewhere) as a way of developing their skills, **practice and experience were considered by all as the most effective way in becoming competent.** [participant] felt “You’ve got to have quite a bit of experience….if you haven’t seen other posts, you can’t really know”. [participant] added, “there was a lot of practice shifts first which was great”. **This practice allowed moderators to receive feedback.** [1]

**“**Training was like **doing practice shifts then receive feedback to develop** rather than formal. There were a few modules but as I was already with ((Name)) it was left for me to do.” Moderator 11

“Yeah I think those were the main things we were learning through our session and it was very supportive because **we would go through the website – the forums and we would be reading what we have typed u**p, what our messages were and we could be like, ‘Oh this was really good. Maybe you could improve this by doing this.’” Moderator 5

Not being trained, well that was difficult because it’s like anyone learning a new job but where e**veryone can see everything – every mistake you’re making and can screenshot every mistake you’re making and there’s nowhere to hide a**nd you have professionals there who you’re taking on their role with no knowledge with them scrutinising not necessarily in a cynical way but there’s a scrutiny there because you’re taking on their role and could get anything wrong and it can be picked up on so it’s very out in the open. Moderator 9

“This **confidence developed over time, through practice** and experience of moderating self-harm, **as moderators had seen and dealt with most scenarios and felt clear about the action required in different situations**.” [1]

O

Although some participants mentioned training (through The Mix or, often, elsewhere) as a way of developing their skills, practice and experience were considered by all as **the most effective way in becoming competent.**  [1]

once they’ve done the e-learning they’re ready to do a practice shift so they do a few of those. Normally they only really need to do like two but it depends, **sometimes people need a bit more practice so we give them three or four shifts until we’re confident that they’re ready to go solo** because they are doing shifts on their own we do a DBS check with them as well. Host 3

Evidence sources:

[1]

Moderator 4

Moderator 5

Moderator 8

Moderator 9

Moderator 11

Moderator 13

Host 2

Host 3

CMOC 7: Moderators feel more able to carry out the role effectively when they receive personalised, co-designed training

Moderation is a complex, difficult job requiring training (C). If training is personalised to the culture and content of the specific forum and co-designed with (and delivered by) people with experience of moderation (M-resource), then it will be is more effective at building competence to moderate well (O) because moderators understand the relevance and applicability of information (M – reasoning).

### Example evidence

C

“have a well-resourced moderating team and don’t underestimate how much input and support they need and I don’t mean that in the sense that they’re needy I mean that in the sense of **it’s actually quite a difficult job**. It sounds quite easy just replying to a few posts but actually it’s a very skilled role and **I think we underestimate how skilled and nuanced and powerful it is so I think it needs training and support for moderators** and space for them to reflect on their work just like therapists would in another context.” Host 5

M

An annual training day for Champions is also held in Melbourne. **Throughout the year, champions contribute to the agenda for this day by providing feedback about desired areas for training.** This feedback is gathered via the private champions forum, and group debriefings. Forum manual 2

“Yeah so we undergo full training and it’s usually about just over an hour’s training session delivered by – m**ine was delivered by a colleague who’s been a [forum name] clinical moderator for many years**…. Each of the service I know because I’ve been trained on all of them though they’re very different and the activity that they’ve had is quite different and potentially you can see the way that other people as in moderators response is quite different per [forum name] and so I’m quite lucky because I have quite a wide view of lots of the [forum name] so moderate across the board so yeah, I think the training it really outlines what your remit is as a moderator.” Moderator 13

“I guess we’ve done a lot of work with our community guidelines so what’s nice is we’ve made like – we call them a **[moderators] group so it’s a small group of 11 active members and they work on projects with us, so they’ve helped rewrite our community guidelines so it fits in with what community members understand in a way that reads in their language** because they were written by someone else years ago. It wasn’t a very friendly language.” Moderator 6

“personalisation would be quite key and may need to take into consideration different factors such as the size of the teams [including: 0:49:40.1] just being one person and also their background so another consideration is the professional versus non-professional forum especially non-professional may be more readily expected to deal with more harmful content, so that’s important to consider and **personalisation I’d say that anything that can be personalised in as many ways as possible would be good because of how varied the moderators are in their experience background** and how it feels there’s not much for the moderators also for themselves but mostly just for **how to be – the practicalities of being a good mod**.” Moderator 9

O

“Yeah so we undergo full training and it’s usually about just over an hour’s training session delivered by – mine was delivered by a colleague who’s been a [forum name] clinical moderator for many years…. Each of the service I know because I’ve been trained on all of them though they’re very different and the activity that they’ve had is quite different and potentially you can see the way that other people as in moderators response is quite different per [forum name] and so I’m quite lucky because I have quite a wide view of lots of the [forum name] so moderate across the board so yeah, **I think the training it really outlines what your remit is as a moderator.**” Moderator 13

## Evidence sources:

Moderator 6

Moderator 9

Moderator 13

Host 5

Forum manual 2

CMOC 8: Moderators feel more able to carry out the role effectively when they hold realistic expectations regarding control

Moderators work within the constraints of anonymous, online forums (C). If moderators keep in mind what is realistic in terms of how much control they have over a users’ situation (M – resource), they can learn to manage unrealistic expectations (M- reasoning) and are less likely to become overwhelmed (O).

### Example evidence

C

**It is important to remember that working on the delivery of a specific health intervention such as [FORUM NAME] will have constraints**. This may be challenging for some people who may be motivated to undertake this type of work through a desire to transform and fix problems. Whilst [FORUM NAME] has the potential to improve the lives of many carers it cannot, on its own, transform every problem in mental health care. Forum manual 1

M

“I think with the moderation sometimes it’s wanting to get people support fast enough and **so you have to remind yourself if a post is waiting a few hours or a day maybe actually it’s pretty average for a CAMHS waiting list just for an assessment to be 18 months.** I know one area currently where to get an ADHD assessment it’s a five year wait.” Moderator 12

“I try and flip reverse it to think that either way that person is sat there with those issues and at least by having this opportunity to share it in an anonymous space they are getting some sort of relief or sense of control over that. They’re able to express where maybe they haven’t been able previous to express or they’ll never be able to express in a face to face situation because they just don’t feel comfortable with that so I **always try and hold on to that small silver lining**.” Moderator 7

“Having to learn to be okay with and accept that other people are going to do what they’re going to do and to not realise that doesn’t mean that I’m okay with some serious things that may happen but that you **just need to accept it sometimes when you don’t have much control over the situation** so it’s been more mindset for me.” Moderator 9

O

“It's human nature to wonder and to sort of want more and sometimes people don't give that or they won't return or you'll think the worst. That can lead you to make assumptions, but, it's just not a space where you can do that, otherwise **it will become too overwhelming, you have to take what you've got in this space**. (Freya)” [4]

## Evidence sources:

[4]

Moderator 7

Moderator 9

Moderator 12

Forum manual 1

CMOC 9: Moderators feel more able to carry out the role effectively when they are given risk-related training and protocols

In the context of an online environment where the potential for risk related posts is high and non-verbal cues are removed (C1), moderators who receive evolving, up-to-date guidance on how to identify and respond to risk appropriately (M – resource) will feel safe to respond to risk related posts (O) because they are less worried about making a mistake in a situation where a user is already at high risk (M – reasoning). Risk protocols may be especially important to health service staff moderating forums where they are familiar with working to protocol and may be concerned about impact on their professional status if no protocols are in place (C2).

### Example evidence

C1

A key difference in computer-mediated communication is the absence of ‘face work’—**non-verbal social cues such as facial expressions and gestures that can influence the interpretation of meaning in communication [references]. The absence of these cues creates key challenges for e-mental health clinicians**. For example, without visual cues **it can be difficult to interpret and understand potentially troubling material** disclosed by clients online, and the relevance of this disclosure to a person’s imminent risk of harm. **[6]**

M

“Our **community guidelines is something that’s ever evolving**…a lot of our young people are very savvy about for example AI and key words like [blocks: 0:12:26.5] on other platforms so they will constantly find ways of talking about topics or trying to talk about topics that don’t meet our boundaries so it is something **that’s very much a live document and constantly updated**.” Moderator 12

[Forum moderators] must identify risks to participants; these risks may be to the participant or another person and for this reason every post on the Forum and Direct Message systems should be read in depth, and in the majority of cases, responded to in a timely manner. When a risk is identified **it is vital to the safety and security of everyone that the Risk protocol is strictly followed**. Forum manual 1

obviously when it comes to things like mental health and higher risk discussions **there’s not a lot of scope to make mistakes** because obviously that impacts that individual who might be in a higher risk situation. Moderator 6

O

I had a lot of sympathy with them because they didn’t have a protocol – so you **need a really clear system that the moderators feel safe** in which is about what should they be doing and if somebody put a risk issue in and something happened what protects the moderator basically.” Host 5

C2

“the forum was going to moderated by **clinical staff ...problem was they were very worried about risk and about are they responsible.** Clinicians spend their lives assessing risk and thoroughly and then making sure if there’s a risk issue they have documented everything, they’ve referred it to the right place etcetera. The NHS is risk obsessed and so in the forum one of them actually said, ‘**I’m not willing to risk my nursing registration because we don’t have a protocol in the trust about how to manage risk on a forum so I’m not doing it,’** and I had a lot of sympathy with them because they didn’t have a protocol” Host 5

## Evidence sources:

[6]

Moderator 5

Moderator 6

Moderator 7

Moderator 10

Moderator 12

Host 5

Forum manual 1

Forum manual 3

CMOC 10: Moderators with limited mental health experience feel more able to carry out the role effectively when they are given mental health literacy training

For moderators with limited mental health experience in relation to the forum content (C) training focused on developing mental health literacy related to the specific mental health experiences likely to be discussed on the forum (M - resource) will enable them to reply to users’ posts with more appropriate responses (O) because they feel more prepared and comfortable to respond to the users’ personal situation (M – reasoning).

### Example evidence

C

“I think **because someone who’s maybe just started in mental health which that was me**, it was having examples of what users could post so yes [scenarios: 0:24:51.9] really and how best to respond because that took a while because **mental health wasn’t my background so it took a while to understand what was appropriate and was the right way to respond** so yeah that probably.” Moderator 4

M

Often, it took the moderators several weeks to become fully comfortable with their duties and **those with a stronger background in mental health reported feeling more prepared**. **[5]**

“I don’t know if I’m contradicting myself but **I would appreciate a bit more time spent reading about the conditions** and the communities that we do have. There are some communities – I didn’t even know we’re a community so I think better knowledge of what actual communities are and what actual conditions that we talk about would be useful just for me personally, I think that would be useful even though…” Moderator 11

There are many forms of mental illness (like anxiety and depression) and there is still a lot we don't know about the root causes of mental illness and our definitions of disorders are frequently changing. **We don't expect you to know them all but will explore a few to ensure your awareness and provide you with a general understanding of the medical perspective of mental illnesses.** Forum manual 3

O

“because **mental health wasn’t my background so it took a while to understand what was appropriate and was the right way to respond**.” Moderator 4

Evidence sources:

[5]

Moderator 4

Moderator 11

Forum manual 3

CMOC 11: Moderators feel more able to carry out the role effectively when they can signpost to host-approved resources

If moderators have access to host-approved up-to-date resources and services in relation to mental health (C) then they will find it less difficult to respond to posts outside their area of expertise (O) because they feel reassured (M –reasoning) by the option to signpost users to other resources (M – resource).

### Example evidence

C

“it’s very vast the resources that they have but essentially anything that might benefit that person rather than trying to find these resources here, there and everywhere or searching things online, the real benefit of [forum name] is that **those resources have been chosen by the service so they’re really service approved information**.” Moderator 13

M

**I’m reassured by the fact that I gave them links** to a bunch of – . Moderator 10

In 22 blog post comments, **moderators provided expertise in the form of either recommending local resources such as crisis centers or education material or sharing professional knowledg**e on a variety of topics related to mental health or use of social media. In 3 comments, users perceived expertise from moderators and responded with acknowledgement and appreciation, including statements such as “these are really helpful.” **[5]**

O

“Because I am a registered nurse and midwife and health visitor on the nursing midwifery council I have to protect – I can only work within my sphere of practice. I can’t be advising about mental health medication for example because **I’m not a mental health nurse so what I would do then if it’s outside my sphere of practice is I would be signposting them** to say, ‘If you’ve got a medication query you could ask your GP, you could ask a pharmacist, you could ask your care coordinator.” Host 3

so we have resources, we have a database and lists of conditions that we allow members to put on their profile and the list of treatments, so I kind of rely on that but yeah it’s **not knowing every illness and every condition, every treatment does make it a bit difficult.** Moderator 10

Evidence sources:

[5]

Moderator 10

Moderator 13

Host 3

CMOC 12: Moderators feel more able to carry out the role effectively when they work as part of a diverse team

Moderators are often exposed to distressing content that they are required to respond to (C). Being able to draw on a team with diverse skills and experience (M – resource) reduces moderator anxiety about responding (O) because they do not feel solely responsible for user well-being and more supported to provide a collaborative response (M – response)

### Example Evidence

C

**“it does hit deep when you have someone saying something like that** so you need to have that team around you so it’s not just you taking on the reading, the decision making and the action on it, all on yourself.” Moderator 6

M

“Each shift has three layers of clinical staff on duty and present. We have **our first layer who we call [moderators]**. Again, all licensed practitioners. The number of [moderators] varies from shift to shift depending on busyness and that kind of thing but they are there to interact with the community anonymously, so ((Name)) entire platform is anonymous and it is enforced anonymity for the community itself. The [moderators] are l**ed by an experienced lead wall guide**. Sometimes two depending on how busy the shift is and the [moderators] responsibilities are broken into different categories depending on the busyness of the shift and then on every shift there is a **senior clinician on duty** and the senior clinician is responsible for the final call on difficult cases, leading the **collaborative decision making** during the shift and then working off platform in what I would call intensive case management in the event that risk is identified.” Host 1

“having a team around you is one of the most important things because it means that **you’re not solely responsible**.” Moderator 10

“having a team on you to second guess, have a **second pair of eyes** so you’re checking on that desensitisation kind of thing and then also **shared responsibility, shared experience and breaking up that responsibility** I guess.” Moderator 6

“I don’t really know who any of the other moderators on the forum are so **we do it in isolation rather than working as a team** but from what I’ve heard from other forums where they are a bit more in a team they are like if you don’t know the answer just ask someone else in the team because s**omeone else probably can come at it from the other experience that you’re lacking and can help you**” **Moderator 9**

O

You can read it and you can act on it but also just taking out that actual decision, just giving that to someone else is **really handy I think mentally** WS1_RJ_Mo_HU_001

**if there’s something on there we were concerned about** we’d share it with each other and say, ‘Ooh, I’ve just noticed when I was on there on Monday that so and so said this. Can you just – what has she said on Tuesday or has anything changed by Wednesday?’ so we do that but maybe not enough. Maybe we need to do a bit more peer support work and check in with each other to see how was it going, WS1_RJ_Mo_Sh1_005_Redacted

## Evidence sources:

[1]

Moderator 2

Moderator 6

Moderator 8

Moderator 9

Moderator 10

Moderator 12

Host 1

Forum manual 1

CMOC 13: Moderators feel less able to carry out the role effectively when they have a conflicting work role

Where moderators have another role, such as a mental health professional delivering in person services (C), and the associated responsibilities of the role conflict with those associated with moderation (M – resource) then moderators will find it tough to fulfil conflicting responsibilities (M – reasoning) and there is a risk that moderation becomes deprioritised (O).

### Example evidence

C

“Yeah I think for me it’s easy because it is my job but **if you’re a clinician and you’ve got a full on case load** and you’ve got staff vacancies as they have at the moment they are on their knees so to ask them to be moderator on [forum name] as well is a big ask” Host 3

M

“It can be **tough** sometimes. I**f the service is really busy then unfortunately sometimes [forum name] can be less active by moderators**. This is something we’re trying to get around because it’s equally as important as our other roles in [forum name]. There’s so much the users can gain from using [forum name] so we want to make sure that it’s getting the support it needs really to keep running.” Moderator 4

“**sometimes it can be quite hard because we’re not given any extra time to do [forum name]. It’s part of our workload** so I could be **out doing visits all day on Monday but I’ve still got to monitor** [forum name] and sometimes it’s quite hard to do because there’s not an app which is something that users struggle with and we struggle with. It’s not an app so it’s not something that we can access very quickly so yeah it’s quite hard to dip into it sometimes to just check in or it could be that I’m out on a visit and someone might say, ‘Oh I’ve noticed that somebody had said something a bit worrying,’ and you’re thinking to yourself, ‘Oh god, it’s going to play on my mind while I’m on a visit,’ and hope that nothing then escalates before I can pop a message in so yeah.” Moderator 4

O

There were lots of other stresses and pressures across the team, staffing issues and things like that I think it has just become accepted that **things will fall off the agenda sometimes** and that’s okay and we’ve got to try and be compassionate towards ourselves and out teams and out colleagues but **it is frustrating when it is always [forum name] that’s getting forgotten about.** Moderator 3

## Evidence sources:

Moderator 3

Moderator 4

Host 3

## CMOC 14: Moderators feel more able to carry out the role effectively when they have the options to explore sensitive topics away from the forum

a) Where users post content to the forum that is sensitive or personal (C) and the forum has a function to message a user privately and directly (M - resource), moderators can offer more specific, user-centred support (O) because they feel comfortable exploring sensitive or personal issues away from the forum (M – reasoning).

### Example evidence

C

“Private messaging (PM) lets you contact Forum users individually instead of replying publicly to a **sensitive or personal message** on a Forum thread." Forum manual 3

M

“**They also had the option to encourage someone to go into direct messagin**g so if they felt that the relative had something – if the relative wanted to direct message they could and also if the [moderator] felt that they’d r**ather have the conversation with somebody off the forum because they felt it wasn’t helpful** for the people they could say, ‘I’m gonna send you a direct message,’ and they **had the ability to take it offline and I think that was actually really helpful**.” Moderator 9

O

it’s normally if they’re worried about someone they want a bit **more support from us and reassurance** that we’re keeping an eye on them and checking in on them. Host 3

## Evidence sources:

Moderator 9

Host 3

Forum manual 3

b) Alternatively, private messaging may encourage users away from the forum and increases the chances of user dependency on moderators (O), which may increase moderator burden (M - reasoning).

### Example evidence

M

Yeah I guess we do see more people they want a bit more one to one support from us from the moderators so we have to do **quite a** **bit of work to close down those conversations** so it doesn’t [have] like a one to one space and **just encourage them to reach back out onto the boards**. Host 3

O

“If I get a direct message from a member, I generally respond to the situation but **gently guide them to use the community.** Reinforce that the platform is a community platform, and they should talk to other members and use the resources on the platform. Make sure they know that this is not a counseling service, and **we do not want to encourage that dependency** unless they are in immediate risk.” [participant] [3]

## Evidence sources:

3

Host 3

## CMOC 15: Moderators feel more able to carry out the role effectively when they have access to technology that support the role

If potentially problematic content is posted (C), and moderators can flag these posts for continued monitoring (M – resource) moderators feel able to respond quickly if there is a need to intervene (M – reasoning) making it easier to meet the demands of the role (O).

### Example evidence

C

“I **usually try to pre-empt topics that can potentially become problematic** (such as sensitive topics related to religion or minority groups), and will flag the posts so we can monitor that post to assess the language quickly and carefully (003)” [3]

M

“I usually try to pre-empt topics that can potentially become problematic (such as sensitive topics related to religion or minority groups), and **will flag the posts so we can monito**r **that post to** **assess the language quickly and carefully** (003)” [3]

The Forum has been set up to **automatically alert moderators about certain things that are posted that might go against the Peer Discussion Forum guidelines**. For instance, alerts will be sent to moderators (including yourself) when users include email addresses and web links in their posts (Flagged by the system using the "@"sign or "www." e.g.) **We notify moderators of these rather than automatically removing them** in case what the user is sharing is genuine and helpful information! For instance, users may want to share a link to a helpful resource - which is okay! If someone shares an email address or inappropriate link - remove it and consider privately messaging the user! Forum manual 3

“it also means you can **monitor it before an escalation** has started so sometimes when someone mentions certain words like religion I can look at the thread and there’ll be absolutely no issue but I can **keep an eye on it which means you can stop before you get the whole massive debate”** Moderator 8

O

“you know, the first comment that crops up which you’re like uh, you can close the post and I think that would be really good for the forum and I think that would also **stop people being like, ‘Oh look, the moderators can’t do their jobs.’** Moderator 8

## Evidence sources:

Moderator 8

Moderator 10

Host 1

Forum manual 4

Forum manual 3

## CMOC 16 Moderators feel more able to carry out the role effectively when user-friendly interaction mechanisms free up moderator time

In online forums where most interactions are post-comment based (C), designing different interaction mechanisms (e.g. hug emoji) for support (M – resource) allows moderators to focus on more detailed text-based posts where necessary (O) because they feel supported by the community to offer matched support to other forum users (M-response).

### Example evidence

Presently, **OMHFs rely on the structure and design of post-comment discussion threads** for provisioning and reaching out for support… By designing d**ifferent interaction mechanisms for reaching out and provisioning social support, we believe the community can better manage support matching**… Such a design can **help in delegating responsibilities that are currently taken up by moderators to other member**s. **[2]**

## Evidence sources:

2

## CMOC 17: Moderators feel more able to carry out the role effectively when they are in the right frame of mind

When moderators are in the right frame of mind i.e. not experiencing significant distress outside the forum and are aware of the risk of desensitisation through repeated exposure (C), familiarity with the forum content through repeated exposure or lived experience (M – resource), increases moderator resilience to the emotional impact of moderating difficult content (M –response) and reduces the likelihood of distress (O).

### Example evidence

C

Moderators were very aware of the emotional demands of moderating self-harm. Most described **moderating only if they had the energy and were in the right frame of mind** Moderator 9

“The best combination was relatives who had been there, had experienced it, had the expertise but maybe **weren’t right in the middle of it in terms of a crisis themselves** so where people were still managing that in a very present way they found it actually quite difficult.” **Host 5**

“Our [capacity: 0:28:51.4] and our experience of risk changes all the time so it can be that you read a post and actually it's something that has personally affected you and so that might hit differently, it might be quite difficult. It might be – I say to people **if you’ve not slept well the night before** you might see a post and think, ‘Why am I crying over this post about a dead hamster?’ but actually if you look at it you’re like well of course I haven’t slept well and maybe **haven’t eaten properly** today so my emotional resilience is lower and maybe things like if you’ve got PMS all sorts of things can change how we view a post so as much as we have a lot of boundaries and rules and policies and procedures a lot of the moderation can be quite subjective.” **Moderator 12**

“I think I handle it quite well. I think **because I’m so used to it**. I think my issue tends to be I have to **make sure I’m not desensitised** but it is something where it doesn’t generally on a personal level upset me.” Moderator 12

“We do have a duty of care for everyone not just for individuals so I think getting that balance was really hard and **you can find yourself going a bit almost desensitised to it when you’re looking at that content so frequently** so you have to have other people involved.” **Moderator 10**

M

“I’m not easily overwhelmed by other people’s difficulties and I’ve **come across these things a lot so for me the personal experience or just seeing a lot of things so it doesn’t affect me** as much as it might potentially some other people so there are elements that surprise me sometimes still but generally it’s not too bad.” Moderator 9

think what we’ve found over the years is actually sometimes **it’s the people without lived experience that are more distressed**. Moderator 12

However, moderators are sometimes individuals who self-harm or who have self-harmed, and **there is a risk of this involvement being triggering for them** too [reference] Where moderators have lived experience of self-harm, i**t was suggested that they should be individuals who have established recovery** and are well supported outside the online group [reference]. **[7]**

“Our [capacity: 0:28:51.4] and our experience of risk changes all the time so it can be that you read a post and actually it's something that has personally affected you and so that might hit differently, it might be quite difficult. It might be – I say to people if you’ve not slept well the night before you might see a post and think, ‘Why am I crying over this post about a dead hamster?’ but actually if you look at it you’re like well of course I haven’t slept well and maybe haven’t eaten properly today so **my emotional resilience is lower** and maybe things like if you’ve got PMS all sorts of things can change how we view a post so as much as we have a lot of boundaries and rules and policies and procedures a lot of the moderation can be quite subjective.” **Moderator 12**

O

think what we’ve found over the years is actually sometimes it’s the people without lived experience that are **more distressed**. Moderator 12

## Evidence sources:

[7]

Moderator 2

Moderator 6

Moderator 9

Moderator 10

Moderator 12

Host 5

Forum manual 2

## CMOC 18: Moderators feel more able to carry out the role effectively when they practice self-care

Moderation can involve exposure to descriptions of potentially highly distressing experiences, such as self-harm (C). Providing moderators with appropriate measures to ensure their well-being (M – resource) can help moderators to feel supported to engage in personalised self-care strategies (M-response) to minimise the negative impact of forum moderation (O).

### Example evidence

C

Moderators described finding moderating self-harm emotionally exhausting, as messages could be shocking” (S1) and distressing” (S3). For some moderators, self-harm was particularly difficult “cause it’s too triggering (V2)” for them. Others described dealing with a lot of self-harm posts as overwhelming, “those posts can be quite heavy and I think you can burn out quite quickly” (S5). [1]

The wellbeing of OMHF moderators is an important aspect that is often ignored. **They read through several posts daily, many of which are psychologically triggering.** These moderators have struggled with some form of mental illness at some point of time, or in the present. In fact, our participant pool included moderators who acknowledged that they felt suicidal in the past. One moderator claimed that they were aware of their own mental health condition, and reckoned that they intentionally should not go through too much of negative content at a single go, and they limited the duration of moderation activities per day. To curb negative outcomes such as **moderators being distressed by reading enormous volumes of negative conten**t, our work implies the need of appropriate measures within OMHF moderation paradigm to ensure their wellbeing. [2]

M

I think also **knowing that it’s okay** for volunteers to take a break during their shift as well. Host 3

“it is something around **taking regular breaks, around knowing what self-care works for you, about feeling able to reach out for support for yourself as well.** It’s something that’s always I think going to be a work in progress but something that I think we’re always really mindful as moderators is that we can be over the course of the day be exposed to a huge amount of very distressing content… if I need a breather I will **step away from my screen. I’ll go and make a cup of tea or stroke the cat**. I’ve known people who have literally done it so that because we all work from home, they’ll leave the house in the morning, **walk around the block and that’s them leaving the house** and when they come back into their house that’s entering the office so things like creating distance. I have an office, I hide my laptop at the end of the day. If I’m on annual leave I do not check my emails, I do not check the forum for example, I have those spaces for myself.” Moderator 12

People in a caring role often put others’ needs before their own and with the additional online [forum] supporter role i**t is vitally important that the supporters develop strategies to look after themselves**. U**nderstanding how to be pro-active about self-care is something that doesn’t need to be done in isolation, and ideally should be at the heart of working practice**. Forum manual 1

The other thing was boundaries about time so something about just coming to work, hearing all this stuff and then going home and how much time do you spend doing it and **how do you make sure you have decompression time so can you do something nice at the end of the day** that isn’t moderating the forum that maybe allows you to switch off a bit before so you’re not going thinking about all the relatives or worrying about all the relatives. Host 5

O

**It is important to recognize the impact that participation in the forums is having on you,** so that you can manage your own level of self-care**.** We want the forums to be a therapeutic, supportive and positive experience for all our members, including our Champions. Forum manual 2

To curb negative outcomes such as moderators being distressed by reading enormous volumes of negative **conten**t, our work implies the **need of appropriate measures within OMHF moderation paradigm to ensure their wellbeing.** [2]

## Evidence sources:

[1]

[2]

Moderator 8

Moderator 10

Moderator 12

Host 3

Host 5

Forum manual 3

Forum manual 2

Forum manual 1

## CMOC 19: Increase engagement by shaping the forum tone

Users of online mental health forums are often going through difficult periods in their lives and may post content that could unintentionally distress other users (C). If moderators are trained on response style and enforcement of rules (M – resource) they can better understand how to work collaboratively to shape the forum in line with the forum culture (M – response) to produce a supportive, productive, judgment-free community (O).

### Example evidence

C

“For example**, if somebody is talking about eating disorders and someone else is coping with an eating disorder symptom we don’t want this person to be bragging about their weight loss through certain symptom engagement and then activating this person**” Host 1

“I think it keeps everything I would say organised if you will or calm. I feel as though if we don’t have moderators a lot could be said – like I said be harmful to that person and it wouldn’t go noticed **especially in a forum full of people who are also going through difficult periods of their lives**.” Moderator 5

M

“For example, if somebody is talking about eating disorders and someone else is coping with an eating disorder symptom we don’t want this person to be bragging about their weight loss through certain symptom engagement and then activating this person so **it’s intended to be supportive and productive** moving towards a healing process and **we work to shape the content collaboratively with the members** to achieve that goal.” Host 1

an important aspect of their work, where they described their **active shaping of a platform** that offers a **destigmatized and judgment-free space** for members to discuss difficult or controversial issues via peer support. [3]

“they also go through a **very extensive internal training shadowing period**, supervised period where we **work to shape all of their interactions into a particular culture so that the [moderators] in particular act as a unified voice in the community that has a very distinct tone that is broadly speaking humanistic interpersonal, supportive, understanding, sympathetic but also maintains the rules**, so we work really hard actually and this is a component in the system that’s hard to explain how **consistent** our clinicians are in the way they engage so that the messaging and the culture and the feel of the community is pervasively supportive and doesn’t have any of the sharp tones and irritability and their interactions are very personalised.” Host 1

O

“This **active ‘shaping force’** brings a **cohesive culture of expressed empathy, sensitivity and care** that helps to deliver **a healthy and safe communit**y” [3]

Evidence sources:

[3]

Moderator 5

Host 1

## CMOC 20: Increase engagement to ensure the forum thrives

Moderators’ responsibilities typically include promoting community activity, for example by welcoming new users and ensuring everyone gets a response (C). When moderators are supported to understand when it is safe to avoid responding themselves (M - resource), they are better able to judge when their input is needed and when it could impede peer support i.e. by ‘jumping in too soon’ and preventing users from connecting with one another (M - response). This leads to a self-reliant community that is enjoyable to moderate (O).

### Example evidence

C

**When a post does not receive sufficient engagement or attention from community members**, [moderators] will often comment on the post themselves, ask questions that elicit other responses from the community or jump start a conversation as an attempt to facilitate the peer support process. [3]

“**every time that someone new comes on the forum to always be as welcoming as possibl**e because you don’t want them to feel that they aren’t welcome on their very first” Moderator 5

M

**Moderators may systematically avoid responding to messages that receive a strong response from the community** or encourage the community to be more self-reliant by withholding intervention **when it is safe to do** so. [8]

“we want in that **time for them to use the time to speak to other mums on there and not just us** really. We’re just there to support and guide and encourage but **really we want them to speak to each other** so actually if there’s a group of mums on there because they’re all [tuned in: 0:06:01.5] at 01:00 in the morning then great, **that’s what we want really**.” Moderator 4

“Just because obviously th**ose people they still have work and there are times where they aren’t going to be exactly when they usually meet up** because they got caught up in something at work or family problems so like you said it’s really being that consistent support throughout the day, throughout the week.” Moderator 5

“I think also as a moderator **speaking to people and responding it makes them come back**. Like I said, we’ve struggled with forum activity and if no one’s replying to their comments they’re less likely to log back in again.” Moderator 8

O

Moderators may systematically avoid responding to messages that receive a strong response from the community or **encourage the community to be more self-reliant** by withholding intervention when it is safe to do so. [8]

“I think it’s the whole allowing community members to see posts that haven’t necessarily been responded to by moderators so **they’re more likely to engage with each other**” Moderator 11

“I also think general forum support by other users is helpful there as well. Sometimes there is no need for me to say anything. **I can literally just read when other people are stepping in and being really supportive**.” Moderator 8

## Evidence sources:

[3]

[8]

[9]

Moderator 4

Moderator 5

Moderator 8

Moderator 11

## CMOC 21: Forming a healthy connection with users

Over extended periods of time moderating on the same forum, moderators may begin to develop relationships with users (C). If moderators identifyand maintain personal and professional boundaries (M - resource) then they are more likely to experience positive relationships with users (O) because they avoid potentially unhealthy dependency of users on them for support and are less likely to worry about users on a personal level (M – response).

### Example evidence

C

“In the beginning you don’t really have any knowledge of anyone on the communities but o**ver time if it’s the same people you almost build relationships because it’s the same people**.” Moderator 11

M

The [moderators] also **ensure that members do not form overly tight bonds with any singular member or with the [moderators] themselves, which can lead to potentially unhealthy dependency**, thus ensuring consistent utilization of peer support between community members. [3]

“Yeah and obviously we don’t have favourites and we try not to get attached but **it’s very hard not to get attached to some of our users** because they are really wonderful young people and we do work with them for a really long period of time sometimes.” Moderator 12

“Another challenge which is kind of working with one of the positives is when professional and personal reasons kind of work together and you hear that someone’s going through an extremely dangerous path it’s very worrying because now it’s like you’re **worrying in a more personal level rather than just keeping it in the professional level** if that makes sense, so it’s also hard to separate them sometimes I would say because of how hard the things are to listen to because they’re going through things that I wouldn’t have thought” Moderator 5

O

“we know our boundaries when it comes to professionalism and our professional lives but sometimes in this line of work especially as a moderator it crosses because **you really do get to know a lot of these people** even though you don’t know their names so it’s just interpersonal skills… It’s really a lot of soft skills that I’ve taken from and I would say personally, a **lot of the users have become such – I would say like friends in the forums as well**. I would say it’s something that’s an **extremely positive thing I got out of it because I’ve heard their storie**s, they talk about their families, their friends, their retirement and it’s just so great to hear about their experiences so at the end of the day moderators are also gaining life lessons from these users and it’s just a positive thing.” Moderator 5

## Evidence sources:

[3]

Moderator 5

Moderator 6

Moderator 11

Moderator 12

## CMOC 22: Using open questions to facilitate connections

When users post messages that do not clearly reveal the kind of support required from moderators (C), asking open questions to find out more (M – resource) decreases moderator anxiety about a user’s situation and increases their ability to provide helpful support (O) because they feel more knowledgeable about the user’s situation (M – response).

### Example evidence

C

“we’ve also learned to read along the lines of what’s being said if that makes sense. They could sound like they’re okay but deep down **they might just want a nudge to kind of get out what they really want to say**” Moderator 5

M

**open questions can be a helpful form of relieving an RS’s anxiety about a situation** and finding out more. For example, asking a direct and specific question such as ‘Do you feel safe?’ whilst not an open ended question, can be important to ask and can very much help assess risk. Forum manual 1

“One of the first things we learned was **learning to give open questions that would encourage the users to come and talk to us more** if that makes sense so never leaving anything as a last – as a close ended questions unless you feel that the conversation has ended, always encouraging them to give – let out as much as they want rather than closing off. That’s one of the first things that we learned.” Moderator 5

O

open questions can be a helpful form of relieving an RS’s anxiety about a situation and **finding out more.** For example, asking a direct and specific question such as ‘Do you feel safe?’ whilst not an open ended question, can be important to ask and can very much help assess risk. Forum manual 1

## Evidence sources:

Moderator 5

Forum manual 1

## CMOC 23: The impact of anonymity on connections

Anonymity can promote the disclosure of distressing experiences, including those related to risk (C). Yet because users’ personal identities are hidden, moderators’ options for offering support and following up on forum users is limited (M - resource), which can lead to disempowerment and uncertainty around user safety (M - response), undermining moderator wellbeing (O).

### Example evidence

C

“at least by having this opportunity to share it in an anonymous space they are getting some sort of relief or sense of control over that. **They’re able to express where maybe they haven’t been able previous to express or they’ll never be able to express in a face to face situation because they just don’t feel comfortable”** Moderator 7

M

“It can be really tricky for the sense that obviously if I’m reading a post that’s really risky and it’s really quite a dark upsetting post to read that can sit with me as a practitioner and I know that **there’s not many steps I can take to support that person** because they’re all real people and I think sometimes with anonymity it can feel like they’re not a real person because obviously we can’t see them, they’re not physically there in front of us, we can’t put a face to a name so I guess in some sense **it can be tricky to then read this post knowing full well that that’s a person that’s sat on their own managing that kind of traumatic experience and you can’t take those steps**” Moderator 7

For example, if being a professional you know details about them, you may be tempted to – you may feel more attached to their story and where there’s anonymity there’s a removal of that to a degree and so sometimes a challenge I’ve had for example, someone had posted essentially saying they were going to take their life and **for a long time I kept checking their profile** Moderator 9

O

“When you hear of these people who have acted on self-harm or taken certain actions we can’t send an ambulance to there – we may know they’re in a high risk situation but we can’t send an ambulance because we don’t know where they live. We don’t even know what country they are in and **people are looking at us like do something about it but we just don’t have the ability, so that’s really challenging I’d say from the emotional aspect** of – the human aspect of knowing someone’s in a really difficult situation and actively not being able to do anything about it is quite hard.” Moderator 10

“We’ve had a trauma specialist in the service before. He said that even he felt that it was kind of like **secondary trauma that we were experiencing at certain times to read in those posts and not being able to take the steps** because as a moderator you’re not in a chat necessarily with somebody or you’re not kind of messaging back and forth with somebody so you could send them a message to check in but you don’t get to see the full step and story of that user. You’re just kind of dealing with post after post.” Moderator 7

### Evidence sources:

[4]

Moderator 7

Moderator 9

Moderator 10

## CMOC 24: Drawing on lived experience to create connections

If a moderator has shared lived experience of the mental health difficulties experienced by forum users (C), they will have a personal, detailed understating of the experiences of forum users (M – resource) enabling then to provide more helpful, emphatic responses (M –response) that best aligns with user needs (O).

### Example evidence

C

“I think yeah p**ersonal experience is important for like most types of forums.** You don’t want to run a football forum if you’ve never played football. **You don’t want to moderate a mental health forum if you don’t have any sort of experience in mental health**.” Moderator 8

M

“All participants felt that **having a detailed understanding of self-harm was an enabler to moderation**. Such knowledge included understanding the spectrum of self-harm, the language around self-harm, and the possible “triggers” and causes of self-harm. **Often this knowledge came from direct experience**, **which participants felt particularly helped their understanding**: “I don’t think unless you’ve experienced it or have worked with people you’d really understand” [participant]. [1]

“I’d say **most people at [forum name] have been affected by bipolar and you can tell and there is that very personal aspect to it** and I think they’ve had moderators in the past that haven’t had that experience and like I said they’re very robotic and perhaps don’t know how to handle situations so yeah I’d say it’s very important to have personal experience.” Moderator 8

“The non-clinical moderators like myself potentially [peers] as well and volunteers **I think we all have had potentially experiences with either healthcare** and had a really good experience and want to give something back or we’ve experienced something with our own or our families mental health that might relate to a particular [Forum Name] so potentially we still have experiences that we can share – **not clinically but it really brings an amazing empathy** ” Moderator 13

O

“I’d say most people at [forum name] have been affected by bipolar and you can tell and there is that very personal aspect to it and I think they’ve had moderators in the past that haven’t had that experience and like I said **they’re very robotic and perhaps don’t know how to handle situations** so yeah I’d say it’s very important to have personal experience.” Moderator 8

**It makes it easier to pass on coping techniques** aside from you know NHS guidelines and stuff. It’s good to say, ‘This is what works for me.’ Moderator 8

### Evidence sources:

[1]

Moderator 8

Moderator 13

Host 2

# **Bibliography**

1. Perowne R, Gutman LM. Barriers and enablers to the moderation of self-harm content for a young person’s online forum. Journal of Mental Health. 2022:1-9. doi: 10.1080/09638237.2022.2069721.

2. Saha K, Ernala SK, Dutta S, Sharma E, De Choudhury M, editors. Understanding Moderation in Online Mental Health Communities. 2020; Cham: Springer International Publishing.

3. Deng D, Rogers T, Naslund JA. The Role of Moderators in Facilitating and Encouraging Peer-to-Peer Support in an Online Mental Health Community: A Qualitative Exploratory Study. Journal of Technology in Behavioral Science. 2023 2023/06/01;8(2):128-39. doi: 10.1007/s41347-023-00302-9.

4. Perry A, Lamont-Mills A, Preez Jd, Plessis Cd. “I Want to Be Stepping in More” - Professional Online Forum Moderators' Experiences of Supporting Individuals in a Suicide Crisis. Frontiers in Psychiatry. 2022 2022-June-13;13. doi: 10.3389/fpsyt.2022.863509.

5. Windler C, Clair M, Long C, Boyle L, Radovic A. Role of moderators on engagement of adolescents with depression or anxiety in a social media intervention: content analysis of web-based interactions. JMIR mental health. 2019;6(9):e13467.

6. Heinsch M, Geddes J, Sampson D, Brosnan C, Hunt S, Wells H, et al. Disclosure of suicidal thoughts during an e-mental health intervention: relational ethics meets actor-network theory. Ethics & Behavior. 2021;31(3):151-70.

7. Abou Seif N, Bastien RJ-B, Wang B, Davies J, Isaken M, Ball E, et al. Effectiveness, acceptability and potential harms of peer support for self-harm in non-clinical settings: systematic review. BJPsych open. 2022;8(1):e28.

8. Milne DN, McCabe KL, Calvo RA. Improving Moderator Responsiveness in Online Peer Support Through Automated Triage. J Med Internet Res. 2019;21(4):e11410. PMID: 31025945. doi: 10.2196/11410.

9. Sindoni MG. ‘#YouCanTalk’: A multimodal discourse analysis of suicide prevention and peer support in the Australian BeyondBlue platform. Discourse & Communication. 2020;14(2):202-21. doi: 10.1177/1750481319890386.
