# Supplementary material for: Understanding the Needs of Moderators in Online Mental Health Forums: Realist Synthesis and Recommendations for Support
Source: JMIR Ment Health. 2025 Sep 26;12:e58891. doi: 10.2196/58891 (PMC12514405; doi:10.2196/58891)
Supplement: Multimedia Appendix 5 [file mental_v12i1e58891_app5.docx]

# Included evidence sources

| **Author & year** | **Country** | **Method (if applicable)** | **Moderator type** | **User type** | **Forum(s)** |
| --- | --- | --- | --- | --- | --- |
| Heinsch et al. (2021) [1] | Australia | Case study on iTreAD trial participant. | Research Clinicians | Young people (aged 18–25) with comorbid depression and binge drinking behaviour. | Private social networking community created specifically for iTreAD research trial. |
| Milne et al. (2019) [2] | Australia | Time series analysis to compare moderator behaviour before and after introducing triage system. | Volunteer peer supporters trained to encourage discussion, and employed staff who deal with distressing content. | Young people (aged 14-25) to seek help and share experiences. | ReachOut.com; a peer support forum for young people which is well-known in Australia. |
| Perowne & Gutman. (2022) [3] | UK | Semi-structured interviews to explore barriers to moderation. | Employed staff. | Young people (aged 16-25) to seek help and share experiences. | The Mix; a charity for young people which runs peer support forums and a telephone service. |
| Windler et al. (2019) [4] | USA | Template analysis of moderator comments, and interviews with moderators. | Research assistants and graduate students. | Young people (aged 14-26) with depression or anxiety. | Supporting Our Valued Adolescents (SOVA); a social networking site developed as part of a wider research study. |
| Saha et al. (2020) [5] | USA | Interviews with 19 moderators from 12 mental health subreddits. | Volunteers. Most were active forum members who progressed to moderators. | People seeking support for a variety of mental health issues. | Twelve subreddits focussed on mental health, including r/depression, r/anxiety, and r/adhd. Reddit is a large forum social network |
| Deng et al. (2023)[6] | USA | Interviews with moderators and thematic content analysis. | Employed staff, who are licensed mental health practitioners. | People seeking mental health support. | Togetherall, previously known as Big White Wall. Digital mental health support commissioned by organisations in the UK, USA and Australia. |
| Perry et al. (2022) [7] | UK, Canada, Australia, New Zealand | Interviews & collective case study, with moderators from three different forums. | Not explicitly stated | People seeking mental health support. | Three forums with focus on providing mental health support. |
| Sindoni (2020) [8] | Australia | Multimodal discourse analysis of BeyondBlue posts. | Not explicitly stated | Australian people struggling with mental health problems. | BeyondBlue; an Australian mental health and wellbeing digital support organisation. |
| Abou Seif (2022) [9] | UK | Systematic review of risks of peer support for self-harm. | Mental health professionals, and people with lived experience of self-harm. | People seeking support for self-harm. Some participants also used the forums with the intention of being triggered to self-harm. | Five self-injury message boards or forums. |
| **Manual** | **Country** | **Method** | **Moderator Type** | **User type** | **Forum(s)** |
| Forum manual 1 | UK | n/a | Employed, based on lived experience. | Adult carers of people with psychosis or bipolar disorder | Forum developed as intervention in a randomised control trial. |
| Forum manual 2 | Australia | n/a | Volunteers who are already active forum members. | Australian people struggling with mental health problems. | An Australian mental health and wellbeing digital support organisation. |
| Forum manual 3 | Canada | n/a | Volunteers who apply for the role online. | Canadians who are experiencing mental health issues and their families who are looking to connect with someone who has gone through a similar experience. | Peer Discussion Forum. |
| Forum manual 4 | UK | n/a | NHS employees | People referred to the forum for mental health support. | Online forums used by NHS trusts in England. |
| Forum manual 5 | Australia | n/a | Volunteer peer supporters trained to encourage discussion, and employed staff who deal with distressing content. | Student (age 14-25) mental health and well-being | A peer support forum which is well-known in Australia. |

References

1. Heinsch M, Geddes J, Sampson D, Brosnan C, Hunt S, Wells H, et al. Disclosure of suicidal thoughts during an e-mental health intervention: relational ethics meets actor-network theory. Ethics & Behavior. 2021;31(3):151-70.

2. Milne DN, McCabe KL, Calvo RA. Improving Moderator Responsiveness in Online Peer Support Through Automated Triage. J Med Internet Res. 2019;21(4):e11410. PMID: 31025945. doi: 10.2196/11410.

3. Perowne R, Gutman LM. Barriers and enablers to the moderation of self-harm content for a young person’s online forum. Journal of Mental Health. 2022:1-9. doi: 10.1080/09638237.2022.2069721.

4. Windler C, Clair M, Long C, Boyle L, Radovic A. Role of moderators on engagement of adolescents with depression or anxiety in a social media intervention: content analysis of web-based interactions. JMIR mental health. 2019;6(9):e13467.

5. Saha K, Ernala SK, Dutta S, Sharma E, De Choudhury M, editors. Understanding Moderation in Online Mental Health Communities. 2020; Cham: Springer International Publishing.

6. Deng D, Rogers T, Naslund JA. The Role of Moderators in Facilitating and Encouraging Peer-to-Peer Support in an Online Mental Health Community: A Qualitative Exploratory Study. Journal of Technology in Behavioral Science. 2023 2023/06/01;8(2):128-39. doi: 10.1007/s41347-023-00302-9.

7. Perry A, Lamont-Mills A, Preez Jd, Plessis Cd. “I Want to Be Stepping in More” - Professional Online Forum Moderators' Experiences of Supporting Individuals in a Suicide Crisis. Frontiers in Psychiatry. 2022 2022-June-13;13. doi: 10.3389/fpsyt.2022.863509.

8. Sindoni MG. ‘# YouCanTalk’: A multimodal discourse analysis of suicide prevention and peer support in the Australian BeyondBlue platform. Discourse & Communication. 2020;14(2):202-21.

9. Abou Seif N, Bastien RJ-B, Wang B, Davies J, Isaken M, Ball E, et al. Effectiveness, acceptability and potential harms of peer support for self-harm in non-clinical settings: systematic review. BJPsych open. 2022;8(1):e28.
