# Supplementary material for: Understanding the Needs of Moderators in Online Mental Health Forums: Realist Synthesis and Recommendations for Support
Source: JMIR Ment Health. 2025 Sep 26;12:e58891. doi: 10.2196/58891 (PMC12514405; doi:10.2196/58891)
Supplement: Multimedia Appendix 6 [file mental_v12i1e58891_app6.docx]

**Multimedia Appendix 6.** Context-mechanism-outcome-configurations (CMOCs).

Where >1 CMOC was developed around the same theory topic, these associated (sometimes rival) theories are presented as separate bullet points under the same CMOC. Where a CMOC had >1 context, mechanism, or outcome, the context, mechanism and/or outcome are numbered consecutively.

| **CMOC Title** | **CMOC** |
| --- | --- |
| **Autonomy (feeling in control of one’s own actions and an alignment between action and personal goals, values, and motivations)** | |
| CMOC 1: alignment of moderation with personal motivation to support others | - If moderators are motivated to support others, perhaps due to previous similar personal experience (context), then they will feel a sense of accomplishment and satisfaction (outcome) from seeing supportive communication and success stories on the forum (mechanism-resource) because they interpret this as evidence that their work has been beneficial in supporting forum users’ well-being (mechanism-reasoning). |
| CMOC 2: conflict in personal motivations | - When users post content that is potentially distressing for the community (context) and moderators are required to edit or remove content (mechanism-resource), this can lead to moderators experiencing discomfort (outcome) because they are conflicted between wanting to respect the needs of the user wanting to share and ensuring the forum does not become a sanitized space versus wanting to protect the community from exposure to potentially harmful material (mechanism-reasoning). |
| CMOC 3: alignment of moderation with expectation for payment | - Where payment is expected within the specific organizational context and of adequate value to the individual moderator (context), payment for moderation (mechanism-resource) may help moderators feel happy and satisfied that their contribution to the forum has been recognized and is valued (mechanism-reasoning 1) and decreases moderators’ perception that they will require additional paid work (mechanism-reasoning 2), increasing their motivation and the likelihood of retention (outcome). |
| **Competence (feeling able to carry out one’s role and being effective in doing so)** | |
| **Increasing competence through initial expectation setting and ongoing support** | |
| CMOC 4: moderators feel more able to carry out the role effectively when they know what to expect | - Moderation can involve exposure to descriptions of potentially highly distressing experiences, such as self-harm (context). Being prewarned of exposure and informed of support available within the team in initial training (mechanism-resource) can help moderators decide (mechanism-reasoning) if or to what extent they are able to moderate at that time (outcome). |
| CMOC 5: moderators feel more able to carry out the role effectively when they are supported by supervisors and peers | - If moderators need support with difficult issues related to forum content or specific users (context) and engage in reflective supervision and peer support in a safe space that fosters trust and openness ( mechanism-resource), then they will be more competent to deal with the issue (outcome) because they feel supported to learn and less isolated in their responsibility for user well-being (mechanism-reasoning). |
| **Increasing competence through training** | |
| CMOC 6: moderators feel more able to carry out the role effectively when they have space and time to practice | - When moderators are given time to build and practice new skills (context), training that involves shadowing more experienced moderators, exposure to the forum, practice shifts, and feedback ( mechanism-resource) increases moderators’ feelings of competence to the moderator alone (outcome) because they feel more confident in how to moderate and less anxious about making mistakes (mechanism-reasoning). |
| CMOC 7: moderators feel more able to carry out the role effectively when they receive personalized, co-designed training | - Moderation is a complex, difficult job requiring training (context). If training is personalized to the culture and content of the specific forum and co-designed with (and delivered by) people with experience of moderation (mechanism-resource), then it will be more effective at building competence to moderate well (outcome) because moderators understand the relevance and applicability of information (mechanism-reasoning). |
| CMOC 8: moderators feel more able to carry out the role effectively when they hold realistic expectations regarding control | - Moderators work within the constraints of anonymous, online forums (context). If moderators keep in mind what is realistic in terms of how much control they have over a user’s situation (mechanism-resource), they can learn to manage unrealistic expectations (mechanism-reasoning) and are less likely to become overwhelmed (outcome). |
| CMOC 9: moderators feel more able to carry out the role effectively when they are given risk-related training and protocols | - In the context of an online environment where the potential for risk-related posts is high and nonverbal cues are removed (context 1), moderators who receive evolving, up-to-date guidance on how to identify and respond to risk appropriately (mechanism-resource) will feel safe to respond to risk-related posts (outcome) because they are less worried about making a mistake in a situation where a user is already at high risk (mechanism-reasoning). Risk protocols may be especially important to health service staff moderating forums where they are familiar with working to protocol and may be concerned about the impact on their professional status if no protocols are in place (context 2). |
| CMOC 10: moderators with limited mental health experience feel more able to carry out the role effectively when they are given mental health literacy training | - For moderators with limited mental health experience in relation to the forum content (context), training focused on developing mental health literacy related to the specific mental health experiences likely to be discussed on the forum (mechanism-resource) will enable them to reply to users’ posts with more appropriate responses (outcome) because they feel more prepared and comfortable to respond to the users’ personal situation (mechanism-reasoning). |
| **Increasing competence through access to resources** | |
| CMOC 11: moderators feel more able to carry out the role effectively when they can signpost to host-approved resources | - If moderators have access to host-approved up-to-date resources and services in relation to mental health (context), then they will find it less difficult to respond to posts outside their area of expertise (outcome) because they feel reassured (mechanism-reasoning) by the option to signpost users to other resources (mechanism-resource). |
| CMOC 12: moderators feel more able to carry out the role effectively when they work as part of a diverse team | - Moderators are often exposed to distressing content that they are required to respond to (context). Having access to a team with diverse skills and experience (mechanism-resource) reduces moderator anxiety about responding (outcome) because they do not feel solely responsible for user well-being and feel more supported to provide a collaborative response (mechanism-response). |
| CMOC 13: moderators feel less able to carry out the role effectively when they have a conflicting work role | - Where moderators have another role, such as a mental health professional delivering in-person services (context), and the associated responsibilities of the role conflict with those associated with moderation (mechanism-resource), then moderators will find it tough to fulfill conflicting responsibilities (mechanism-reasoning) and there is a risk that moderation becomes deprioritized (outcome). |
| **Increasing competence through forum design** | |
| CMOC 14: moderators feel more able to carry out the role effectively when they have the option to explore sensitive topics away from the forum | - Where users post content to the forum that is sensitive or personal (context) and the forum has a function to message a user privately and directly (mechanism-resource), moderators can offer more specific, user-centered support (outcome) because they feel comfortable exploring sensitive or personal issues away from the forum (mechanism-reasoning). - Alternatively, private messaging may encourage users away from the forum and increase the chances of user dependency on moderators (outcome 2), which may increase moderator burden (mechanism-reasoning 2). |
| CMOC 15: moderators feel more able to carry out the role effectively when they have access to technology that supports the role | - If potentially problematic content is posted (context) and moderators can flag these posts for continued monitoring (mechanism-resource), moderators feel able to respond quickly if there is a need to intervene (mechanism-reasoning), making it easier to meet the demands of the role (outcome). |
| CMOC 16: moderators feel more able to carry out the role effectively when user-friendly interaction mechanisms reduce their workload and free up time | - In online forums where most interactions are post-comment based (context), designing different interaction mechanisms (eg, hug emoji) for support (mechanism-resource) allows moderators to focus on more detailed text-based posts where necessary (outcome) because they feel supported by the community to offer matched support to other forum users (mechanism-response). |
| **Increasing competence by supporting well-being** | |
| CMOC 17: moderators feel more able to carry out the role effectively when they are in the right frame of mind | - When moderators are in the right frame of mind, that is, not experiencing significant distress outside the forum, and are aware of the risk of desensitization through repeated exposure (context), familiarity with the forum content through repeated exposure or lived experience (mechanism-resource) increases moderator resilience to the emotional impact of moderating difficult content (mechanism-response) and reduces the likelihood of distress (outcome). |
| CMOC 18: increase moderators’ ability to do the job (competence) through self-care | - Moderation can involve exposure to descriptions of potentially highly distressing experiences, such as self-harm (context). Providing moderators with appropriate measures to ensure their well-being (mechanism-resource) can help moderators to feel supported to engage in personalized self-care strategies (mechanism-response) to minimize the negative impact of forum moderation (outcome). |
| **Relatedness** |  |
| **Supporting users to connect with the forum and each other** | |
| CMOC 19: increase engagement by shaping the forum’s tone | - Users of online mental health forums are often going through difficult periods in their lives and may post content that could unintentionally distress other users (context). If moderators are trained in response style and enforcement of rules (mechanism-resource), they can better understand how to work collaboratively to shape the forum in line with the forum culture (mechanism-response) to produce a supportive, productive, judgment-free community (outcome). |
| CMOC 20: increase engagement to ensure the forum thrives | - Moderators’ responsibilities typically include promoting community activity, for example, by welcoming new users and ensuring everyone gets a response (context). When moderators are supported to understand when it is safe to avoid responding themselves (mechanism-resource), they are better able to judge when their input is needed and when it could impede peer support, that is, by “jumping in too soon” and preventing users from connecting with one another (mechanism-response). This leads to a self-reliant community that is enjoyable to moderate (outcome). |
| **Making a connection with users** | |
| CMOC 21: forming a healthy connection with users | - Over extended periods of moderating on the same forum, moderators may begin to develop relationships with users (context). If moderators identify and maintain personal and professional boundaries (mechanism-resource), then they are more likely to experience positive relationships with users (outcome) because they avoid potentially unhealthy dependency of users on them for support and are less likely to worry about users on a personal level (mechanism-response). |
| CMOC 22: using open questions to facilitate connections | - When users post messages that do not clearly reveal the type of support required from moderators (context), asking open questions to find out more (mechanism-resource) decreases moderator anxiety about a user’s situation and increases their ability to provide helpful support (outcome) because they feel more knowledgeable about the user’s situation (mechanism-response). |
| CMOC 23: the impact of anonymity on connections | - Anonymity can promote the disclosure of distressing experiences, including those related to risk (context). Nevertheless, because users’ personal identities are hidden, moderators’ options for offering support and following up on forum users are limited (mechanism-resource), which can lead to disempowerment and uncertainty around user safety (mechanism-response), undermining moderator well-being (outcome). |
| CMOC 24: drawing on lived experience to create connections | - If a moderator has shared lived experience of the mental health difficulties experienced by forum users (context), they will have a personal, detailed understanding of the experiences of forum users (mechanism-resource), enabling them to provide more helpful, emphatic responses (mechanism-response) that best align with user needs (outcome). |
